# Supplementary material for: Progressive senescence programs induce intrinsic vulnerability to aging-related female breast cancer
Source: Nat Commun. 2024 Jun 17;15:5154. doi: 10.1038/s41467-024-49106-2 (PMC11183265; doi:10.1038/s41467-024-49106-2)
Supplement: Supplementary file 1 — Supplementary Information [file 41467_2024_49106_MOESM1_ESM.pdf]

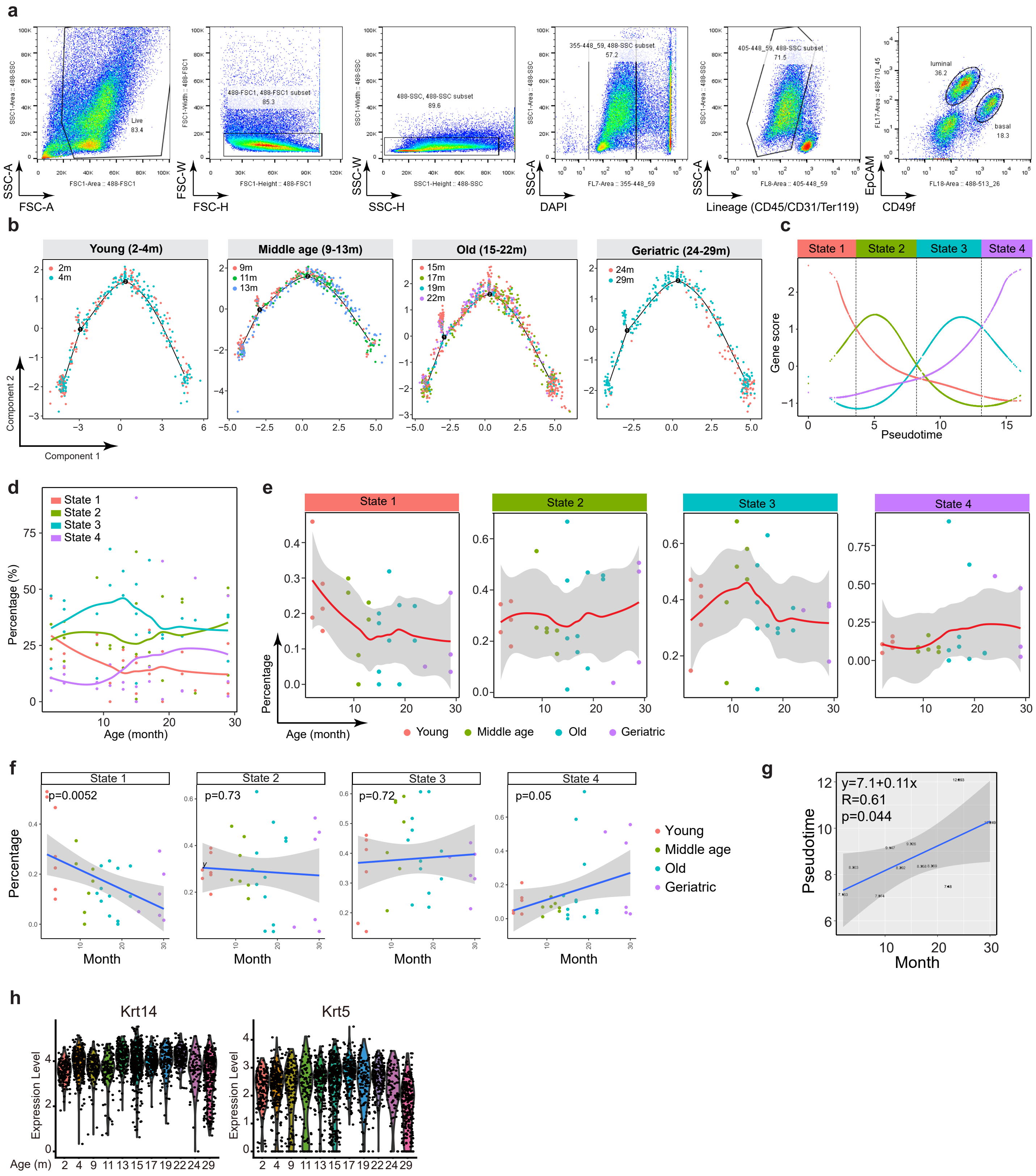
a
Live
83.4
488-FSC1, 488-FSC1 subset
85.3
488-SSC, 488-SSC subset
89.6
355-448_59, 488-SSC subset
57.2
405-448_59, 488-SSC subset
71.5
luminal
36.2
basal
18.3
SSC-A
FSC-A
FSC-W
FSC-H
SSC-W
SSC-H
SSC-A
DAPI
SSC-A
Lineage (CD45/CD31/Ter119)
EpCAM
CD49f
b
Young (2-4m)
2m
4m
Middle age (9-13m)
9m
11m
13m
Old (15-22m)
15m
17m
19m
22m
Geriatric (24-29m)
24m
29m
Component 2
Component 1
c
State 1
State 2
State 3
State 4
Gene score
Pseudotime
d
State 1
State 2
State 3
State 4
Percentage (%)
Age (month)
e
State 1
State 2
State 3
State 4
Percentage
Age (month)
Young
Middle age
Old
Geriatric
f
State 1
p=0.0052
State 2
p=0.73
State 3
p=0.72
State 4
p=0.05
Percentage
Month
Young
Middle age
Old
Geriatric
g
y=7.1+0.11x
R=0.61
p=0.044
Pseudotime
Month
h
Krt14
Krt5
Expression Level
Age (m)
2 4 9 11 13 15 17 19 22 24 29

Supplementary Fig. 1 | Distribution of mammary cells over pseudotime trajectory and age progression.

a, FACS sorting strategy of CD49f^{high}EpCAM^{low} cells. Cell numbers are listed in the Supplementary Data 1.

b, Pseudotemporal ordering of mammary cells from various age groups showing that each age group contains all the cell states. Cells are labeled by colors according to their biological ages in each age group.

c, Expression dynamics of the signature gene clusters of each cell state over the pseudotime trajectory. The boundary of each cell state was determined by the intersections of gene scores from neighboring cell states.

d-e, Dynamics of the proportion of each cell state over the real chronological age.

f, The statistics of the dynamics of various mammary cell states.

g, The correlation between mouse age and pseudotime.

h, Basal cell specific genes (Keratin 5 and Keratin 14) expression in various mouse ages.

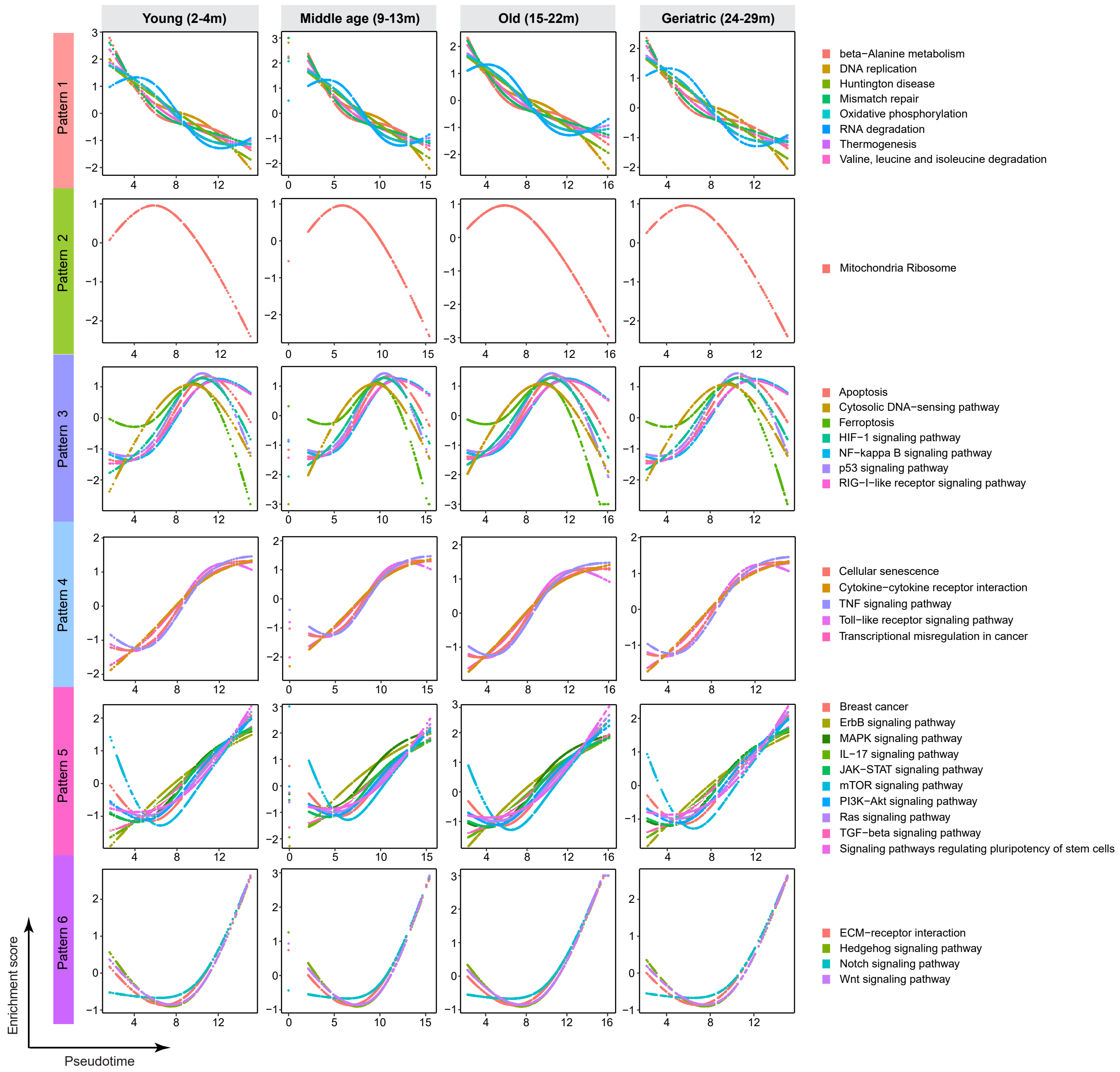

Young (2-4m)
Middle age (9-13m)
Old (15-22m)
Geriatric (24-29m)
Pattern 1
Pattern 2
Pattern 3
Pattern 4
Pattern 5
Pattern 6
beta−Alanine metabolism
DNA replication
Huntington disease
Mismatch repair
Oxidative phosphorylation
RNA degradation
Thermogenesis
Valine, leucine and isoleucine degradation
Mitochondria Ribosome
Apoptosis
Cytosolic DNA−sensing pathway
Ferroptosis
HIF−1 signaling pathway
NF−kappa B signaling pathway
p53 signaling pathway
RIG−I−like receptor signaling pathway
Cellular senescence
Cytokine−cytokine receptor interaction
TNF signaling pathway
Toll−like receptor signaling pathway
Transcriptional misregulation in cancer
Breast cancer
ErbB signaling pathway
MAPK signaling pathway
IL−17 signaling pathway
JAK−STAT signaling pathway
mTOR signaling pathway
PI3K−Akt signaling pathway
Ras signaling pathway
TGF−beta signaling pathway
Signaling pathways regulating pluripotency of stem cells
ECM−receptor interaction
Hedgehog signaling pathway
Notch signaling pathway
Wnt signaling pathway
Enrichment score
Pseudotime

Supplementary Fig. 2 | Pathway dynamics of mammary cells from each age group over the pseudotime.

Enrichment score of pathways from 6 different patterns for young, middle age, old and geriatric mammary cells. Note: the dynamic pattern of each signaling pathway for mammary cells from different age group remains largely the same. This indicates that the dynamic pattern of signaling pathways are determined by cell state instead of the real chronological age. Cell numbers are listed in the Supplementary Data 1.

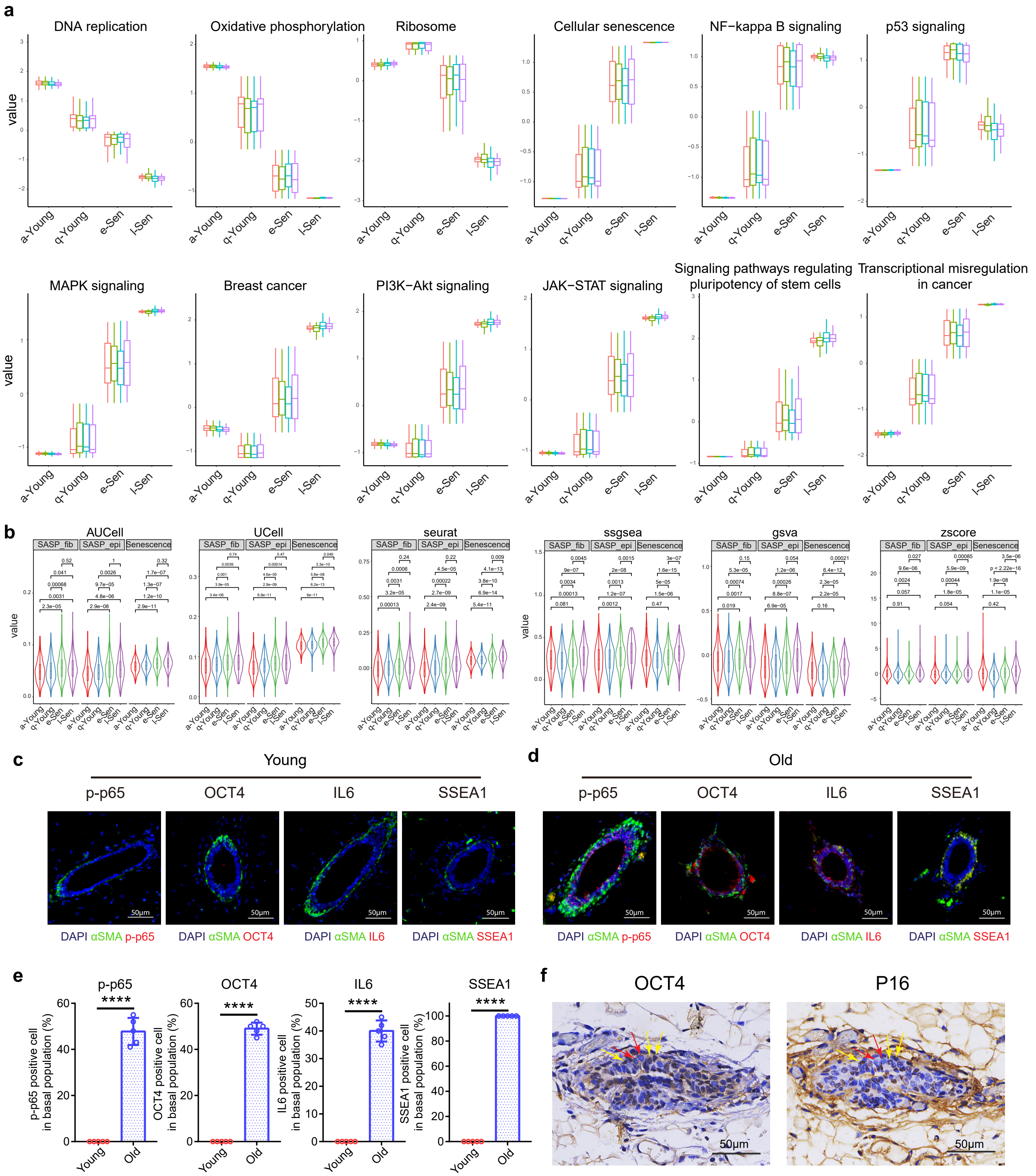
a
DNA replication
Oxidative phosphorylation
Ribosome
Cellular senescence
NF−kappa B signaling
p53 signaling
MAPK signaling
Breast cancer
PI3K−Akt signaling
JAK−STAT signaling
Signaling pathways regulating pluripotency of stem cells
Transcriptional misregulation in cancer
value
a-Young
q-Young
e-Sen
l-Sen
b
AUCell
UCell
seurat
ssgsea
gsva
zscore
SASP_fib
SASP_epi
Senescence
c
Young
d
Old
p-p65
OCT4
IL6
SSEA1
50μm
DAPI αSMA p-p65
DAPI αSMA OCT4
DAPI αSMA IL6
DAPI αSMA SSEA1
e
p-p65 positive cell in basal population (%)
OCT4 positive cell in basal population (%)
IL6 positive cell in basal population (%)
SSEA1 positive cell in basal population (%)
****
Young
Old
f
OCT4
P16

Supplementary Fig. 3 | The aging related pathways in various mammary cell states and mouse ages.

a, Signaling pathway analysis within cellular states in various mouse ages. Cell numbers are listed in the Supplementary Data 1.

b, The aging related pathways in various mammary cell states. Cell numbers are listed in the Supplementary Data 1.

c, Representative immunofluorescence staining of p- p65, Oct4, Il6 and Ssea1 (scale bar, 50μm) in mammary cells from young mice.

d, Representative immunofluorescence staining of p-p65, Oct4, Il6 and Ssea1 (scale bar, 50μm) in mammary cells from old mice.

e, Quantification of p-p65, Oct4, Il6 and Ssea1 in young and old mammary glands. Statistical analysis was performed using two-tailed unpaired t-tests; n=5 samples; data are presented as mean ± SD; ****P<0.0001.

f. Representative immunohistochemistry staining of OCT4 and p16 in the 24 month old consecutive mammary sections. Red arrows point to the two corresponding cells in the consecutive sections negative for both p16 and OCT4. Yellow arrows showed mammary cells positive for both p16 and OCT4.

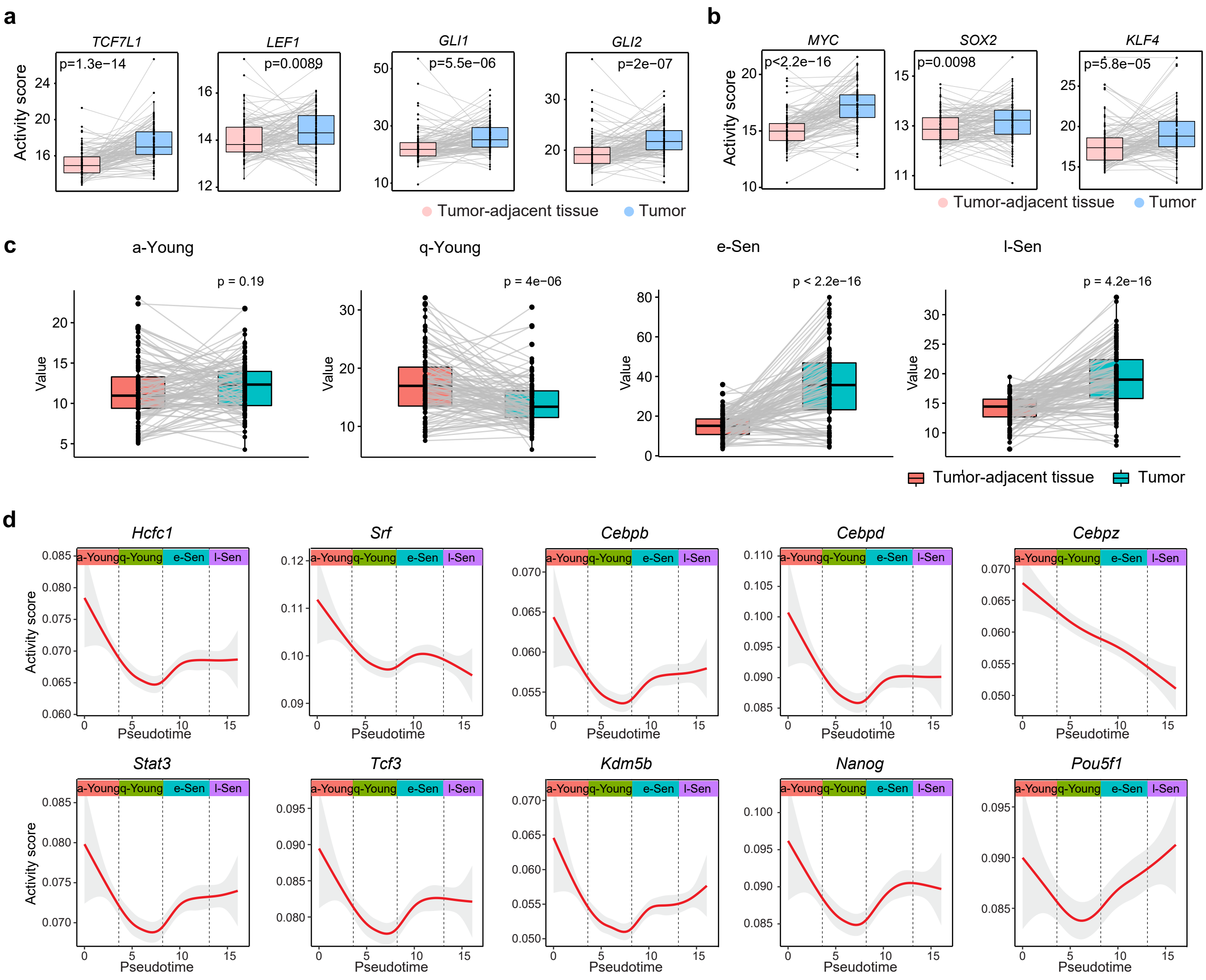
a
TCF7L1
p=1.3e−14
LEF1
p=0.0089
GLI1
p=5.5e−06
GLI2
p=2e−07
Activity score
Tumor-adjacent tissue
Tumor
b
MYC
p<2.2e−16
SOX2
p=0.0098
KLF4
p=5.8e−05
Activity score
Tumor-adjacent tissue
Tumor
c
a-Young
p = 0.19
q-Young
p = 4e−06
e-Sen
p < 2.2e−16
l-Sen
p = 4.2e−16
Value
Tumor-adjacent tissue
Tumor
d
Hcfc1
Srf
Cebpb
Cebpd
Cebpz
Stat3
Tcf3
Kdm5b
Nanog
Pou5f1
a-Young
q-Young
e-Sen
l-Sen
Activity score
Pseudotime

Supplementary Fig. 4 | Activity score of transcription factors enriched in a/q-Young and l-Sen states.

a-b, Wnt and Notch pathway (**a**), stemness (**b**) related transcription factor activity was upregulated in tumor tissue. Statistical analysis was performed using two-tailed paired t-tests.

c, Cell state signature in tumor-adjacent tissue and tumor tissues. Statistical analysis was performed using two-tailed paired t-tests.

d, Activity score of transcription factors enriched in a/q-Young and l-Sen states presented by target gene score over the pseudotime. The aging related pathways in various mammary cell states.

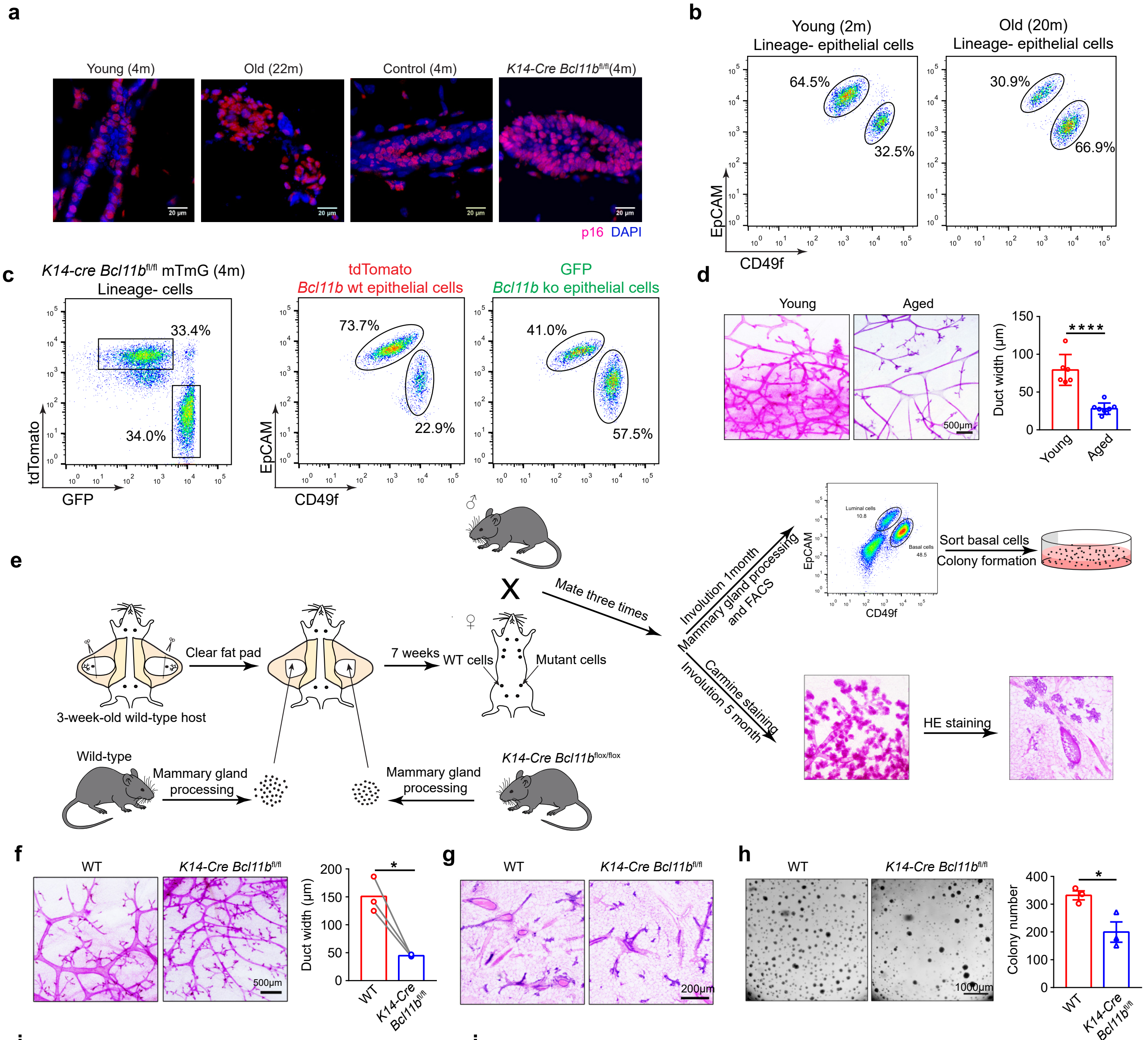

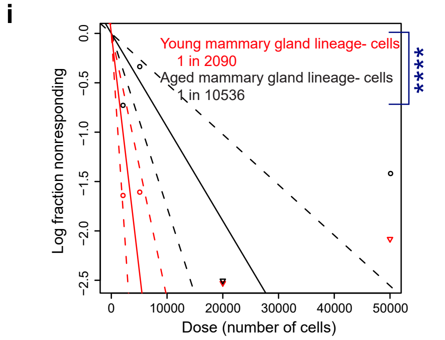

j

Limiting Dilution Transplant of Young and Aged Mammary Gland Lineage- cells

	No. of Injected Cells/Fat Pad	Mammary Tree Outgrowth/No. of Injected Fat Pad	MRU frequency (95% Confidence Interval)
3 month old	50k	4 of 4	1 in 2090 (1 in 1173 to 3724)
	20k	6 of 6	
	5k	8 of 10	
	2k	8 of 10	
18 month old	50k	3 of 4	1 in 10536 (1 in 5675 to 19563)
	20k	6 of 6	
	5k	3 of 10	
	2k	5 of 10	

Supplementary Fig. 5 | Accelerated stem cell exhaustion during ageing and after *Bcl11b* knockout.

a, Representative immunofluorescence staining of $p16^{Ink4a}$ (scale bar, 20µm) in mammary epithelial cells from young (4 month), old (22 month), control *K14-Cre Bcl11b*$^{wt/wt}$ (4 month) and *K14-Cre Bcl11b*$^{fl/fl}$ (4 month) mice.

b, Representative FACS plots of basal cells and luminal cells in young (2m) and old (20m) mammary gland lineage- population.

c, FASC analysis of mammary epithelia in *K14-Cre Bcl11b*$^{fl/fl}$ *mTmG* mice showing the relative proportion of basal cells and luminal cells.

d, Representative wholemount staining and duct width quantifications of young (2m-5m, n=6 samples) and aged (12m- 29m, n=8 samples) mammary glands. Scale bar, 500µm; statistical analysis was performed using two-tailed unpaired t-tests; data are presented as mean ± SD; ****P<0.0001.

e, Schematic diagram for the workflow of the ageing phenotype quantification for WT and *K14-Cre Bcl11b*$^{fl/fl}$ mammary cells after multiple rounds of reproduction cycles. Basically, WT and *K14-Cre Bcl11b*$^{fl/fl}$ mammary cells were transplanted onto cleared fat pads. Recipient mice were mated with male mice for 3 rounds. Whole mount and colony formation assays were performed after the last round of pregnancy.

f-h, Representative pictures of mammary wholemounts (**f**; Scale bar, 500µm, p=0.0045), HE staining (**g**; Scale bar, 200µm) and colony formation (**h**; Scale bar, 1000µm, p=0.0295) of WT and *K14-Cre Bcl11b*$^{fl/fl}$ transplanted mammary glands after multiple rounds of pregnancy. Statistical analysis was performed using two-tailed unpaired t-tests; n=3 samples for each group; data are presented as mean ± SD.

i, ELDA plot of limiting dilution transplant of young (3m) and old (18m) mammary gland lineage- cells. P<0.0001.

j, Table for limiting dilution transplant of young (3month) and old (18 month) mammary gland lineage- cells.

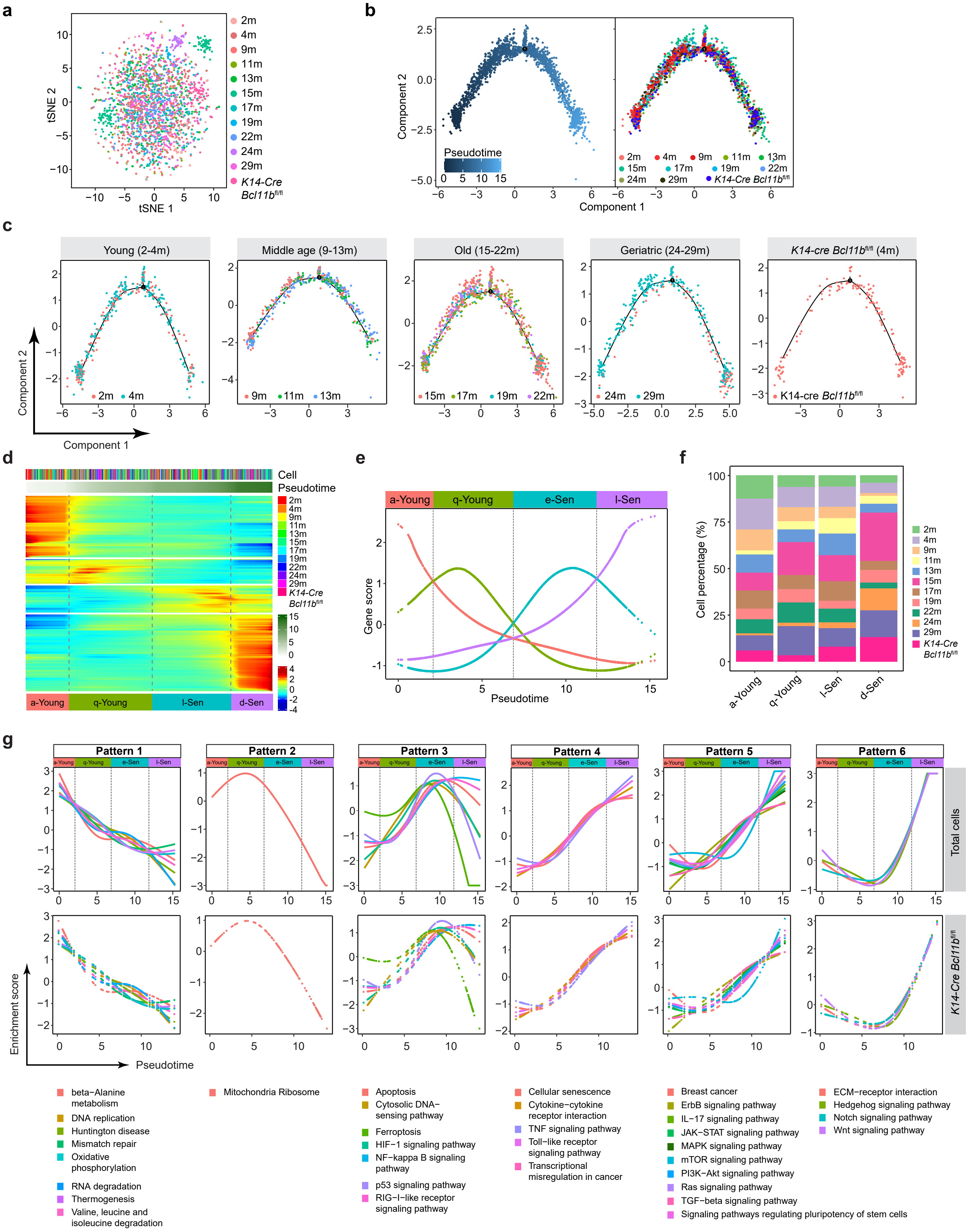
a
tSNE 1
tSNE 2
2m
4m
9m
11m
13m
15m
17m
19m
22m
24m
29m
K14-Cre Bcl11b^fl/fl
b
Component 1
Component 2
Pseudotime
c
Young (2-4m)
Middle age (9-13m)
Old (15-22m)
Geriatric (24-29m)
K14-cre Bcl11b^fl/fl (4m)
Component 1
Component 2
d
Cell
Pseudotime
a-Young
q-Young
l-Sen
d-Sen
e
a-Young
q-Young
e-Sen
l-Sen
Gene score
Pseudotime
f
Cell percentage (%)
a-Young
q-Young
l-Sen
d-Sen
g
Pattern 1
Pattern 2
Pattern 3
Pattern 4
Pattern 5
Pattern 6
Total cells
K14-Cre Bcl11b^fl/fl
Enrichment score
Pseudotime
beta−Alanine metabolism
DNA replication
Huntington disease
Mismatch repair
Oxidative phosphorylation
RNA degradation
Thermogenesis
Valine, leucine and isoleucine degradation
Mitochondria Ribosome
Apoptosis
Cytosolic DNA−sensing pathway
Ferroptosis
HIF−1 signaling pathway
NF−kappa B signaling pathway
p53 signaling pathway
RIG−I−like receptor signaling pathway
Cellular senescence
Cytokine−cytokine receptor interaction
TNF signaling pathway
Toll−like receptor signaling pathway
Transcriptional misregulation in cancer
Breast cancer
ErbB signaling pathway
IL−17 signaling pathway
JAK−STAT signaling pathway
MAPK signaling pathway
mTOR signaling pathway
PI3K−Akt signaling pathway
Ras signaling pathway
TGF−beta signaling pathway
Signaling pathways regulating pluripotency of stem cells
ECM−receptor interaction
Hedgehog signaling pathway
Notch signaling pathway
Wnt signaling pathway

Supplementary Fig. 6 | Single cell ageing trajectory analysis of WT (2-29 months) with *Bcl11b* KO CD49f^{high}EpCAM^{low} cells (4months).

a-b, t-SNE plot showing the clustering analysis **(a)** and the pseudotime ordering analysis **(b)** of scRNA-seq data from young, middle age, old, geriatric mammary cells along with *K14-Cre Bcl11b*$^{fl/fl}$ (4month) CD49f^{high}EpCAM^{low} cells. Cell origins are labeled by colors. Cell numbers are listed in the Supplementary Data 1.

c, Pseudotemporal ordering of mammary cells from various age groups showing that each age group contains all the cell states. Cells of different ages are labeled by colors in each age group. **d,** Heatmap visualization of the dynamic gene expression changes over the pseudotime.

e, Score of differentially expressed genes in each re-analyzed stage along the pseudotime. Definition of a state is based on the intersection point of the neighboring cell states.

f, Relative proportion of mammary cells from various ages of WT mice and *Bcl11b* KO (4month) in each re-analyzed cell state.

g, Activity score of each signaling pathway over the pseudotime of WT mammary cells and *K14-Cre Bcl11b*$^{fl/fl}$ cells. Note: the dynamic pattern of each signaling pathway for mammary cells from different age group and *K14-Cre Bcl11b*$^{fl/fl}$ group remains largely the same.

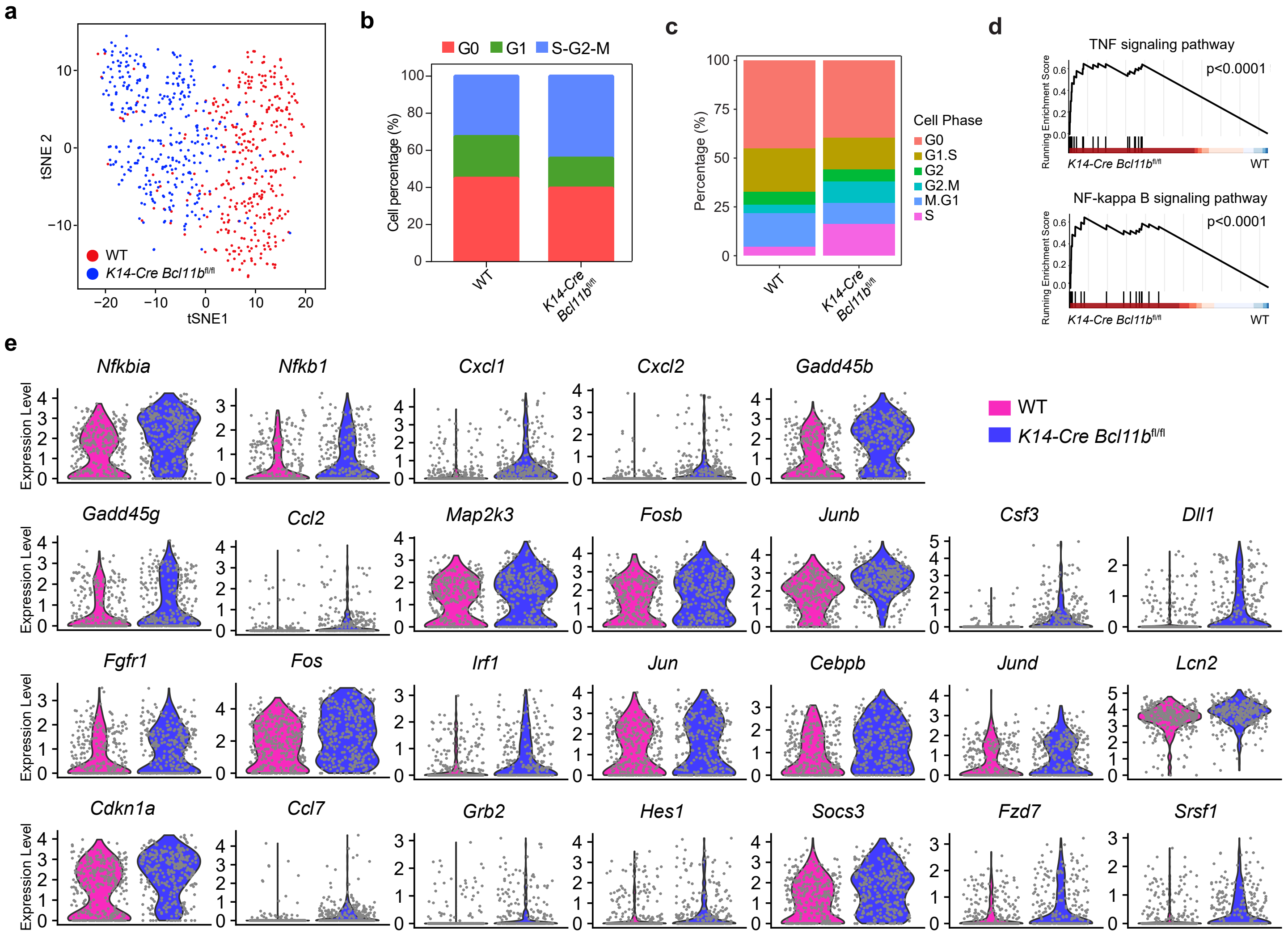

a
tSNE 2
tSNE1
WT
K14-Cre Bcl11b$^{fl/fl}$
b
G0
G1
S-G2-M
Cell percentage (%)
WT
K14-Cre Bcl11b$^{fl/fl}$
c
Percentage (%)
Cell Phase
G0
G1.S
G2
G2.M
M.G1
S
WT
K14-Cre Bcl11b$^{fl/fl}$
d
TNF signaling pathway
p<0.0001
Running Enrichment Score
K14-Cre Bcl11b$^{fl/fl}$
WT
NF-kappa B signaling pathway
p<0.0001
Running Enrichment Score
K14-Cre Bcl11b$^{fl/fl}$
WT
e
Nfkbia
Nfkb1
Cxcl1
Cxcl2
Gadd45b
WT
K14-Cre Bcl11b$^{fl/fl}$
Gadd45g
Ccl2
Map2k3
Fosb
Junb
Csf3
Dll1
Fgfr1
Fos
Irf1
Jun
Cebpb
Jund
Lcn2
Cdkn1a
Ccl7
Grb2
Hes1
Socs3
Fzd7
Srsf1
Expression Level

Supplementary Fig. 7 | Differentially expressed genes in WT and *K14-Cre Bcl11b*$^{\mathrm{fl/fl}}$ CD49f^{high}EpCAM^{low} mammary cells.

a, t-SNE plot showing the scRNA-seq data from WT (red; 4m, 236 cells) and *Kr14-Cre Bcl11b*$^{\mathrm{fl/fl}}$ (blue; 4m, 149 cells) CD49f^{high}EpCAM^{low} mammary cells.

b, Cell cycle fraction analysis of G0, G1, S-G2-M in WT and *K14-Cre Bcl11b*$^{\mathrm{fl/fl}}$ groups.

c, Relative cell cycle proportion in WT and *K14-Cre Bcl11b*$^{\mathrm{fl/fl}}$ groups.

d, Gene set enrichment analysis (GSEA) enrichment analysis of scRNA-seq data from WT and *K14-Cre Bcl11b*$^{\mathrm{fl/fl}}$ CD49f^{high}EpCAM^{low} cells showing the enrichment of TNF and NF-kB signaling pathways in *Bcl11b* KO mammary cells. GSEA from clusterProfiler package was used to perform analysis.

e, Differentially expressed genes in WT and *K14-Cre Bcl11b*$^{\mathrm{fl/fl}}$ CD49f^{high}EpCAM^{low} cells.

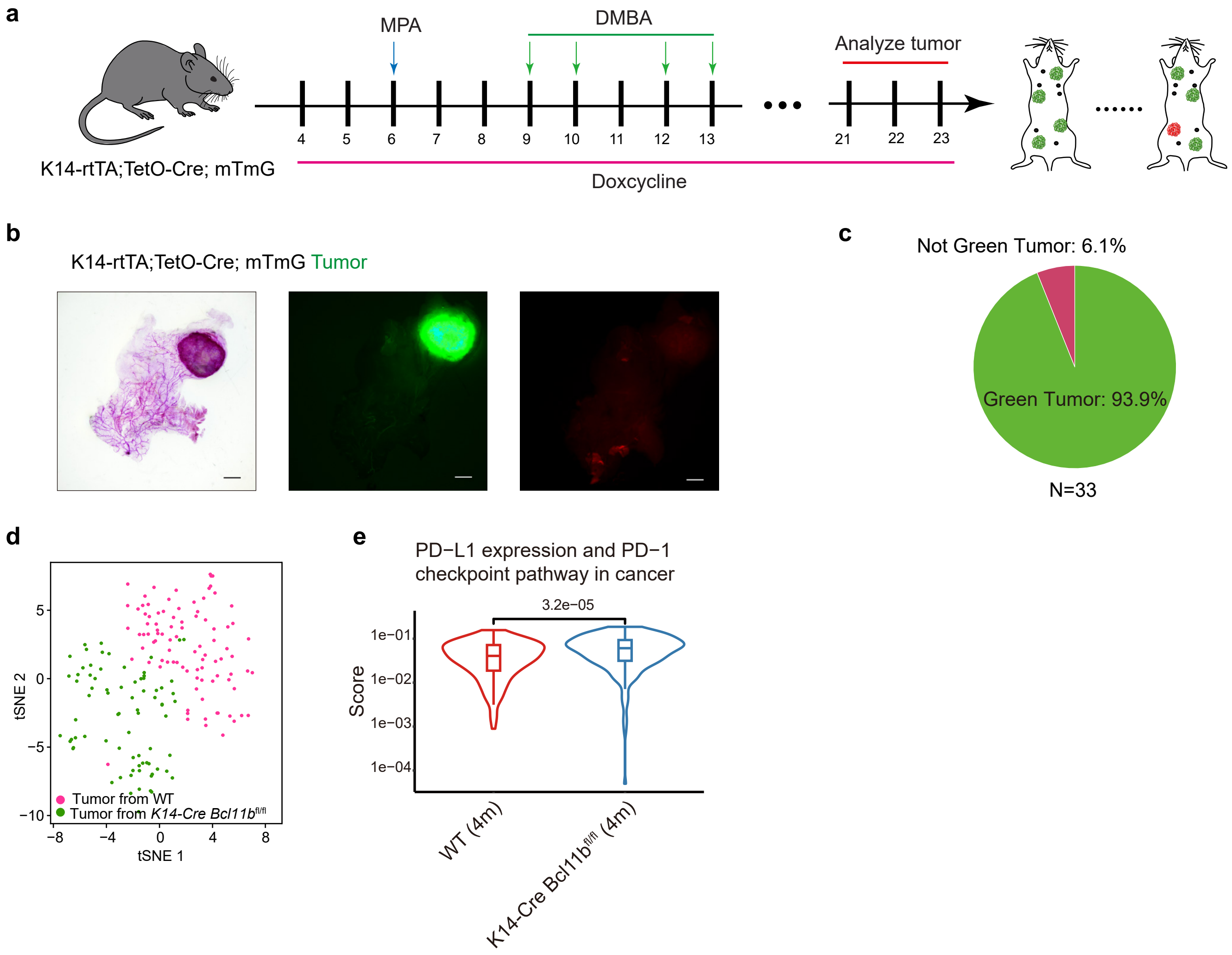

a
MPA
DMBA
Analyze tumor
4 5 6 7 8 9 10 11 12 13 21 22 23
K14-rtTA;TetO-Cre; mTmG
Doxcycline
b
K14-rtTA;TetO-Cre; mTmG Tumor
c
Not Green Tumor: 6.1%
Green Tumor: 93.9%
N=33
d
Tumor from WT
Tumor from K14-Cre Bcl11b fl/fl
tSNE 1
tSNE 2
e
PD−L1 expression and PD−1 checkpoint pathway in cancer
3.2e−05
Score
1e−01
1e−02
1e−03
1e−04
WT (4m)
K14-Cre Bcl11b fl/fl (4m)

Supplementary Fig. 8 | DMBA induced mouse breast tumors mainly originate from basal cells.

a, Basal cells of Krt14rtTA-TetOcre-mTmG mice were labeled with doxycycline induction, and mice were treated with DMBA.

b, Representative tumor images of Krt14rtTA-TetOcre-mTmG mice induced with doxycycline.

c, Green tumor percentage in the outgrowth tumors (N=33 tumor).

d, The t-SNE plot showing the clustering of CD49f^{high}EpCAM^{low} cells from DMBA induced WT (93 cells) or *K14-Cre Bcl11b*$^{fl/fl}$ (77 cells) tumor.

e, PD-L1 expression and PD-1 checkpoint pathway score in WT (93 cells) and *K14-Cre Bcl11b*$^{fl/fl}$ (77 cells) CD49f^{high}EpCAM^{low} cells. Two-sided wilcoxon -test was used.

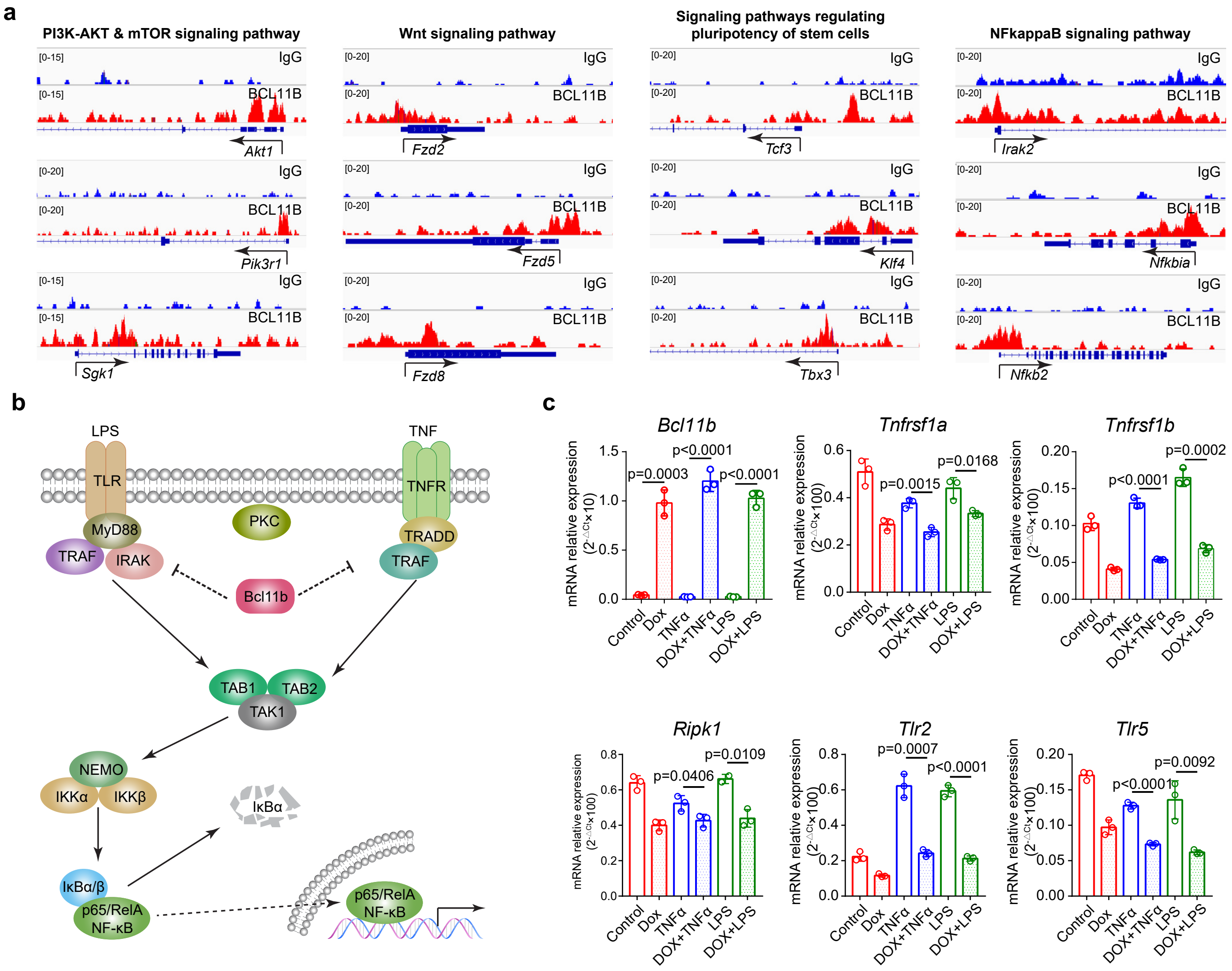
a
PI3K-AKT & mTOR signaling pathway
Wnt signaling pathway
Signaling pathways regulating pluripotency of stem cells
NFkappaB signaling pathway
[0-15]
[0-20]
IgG
BCL11B
Akt1
Pik3r1
Sgk1
Fzd2
Fzd5
Fzd8
Tcf3
Klf4
Tbx3
Irak2
Nfkbia
Nfkb2
b
LPS
TNF
TLR
TNFR
MyD88
PKC
TRADD
TRAF
IRAK
Bcl11b
TAB1
TAB2
TAK1
NEMO
IKKα
IKKβ
IκBα
IκBα/β
p65/RelA
NF-κB
c
Bcl11b
Tnfrsf1a
Tnfrsf1b
Ripk1
Tlr2
Tlr5
mRNA relative expression
(2^-ΔCt×10)
(2^-ΔCt×100)
p=0.0003
p<0.0001
p<0.0001
p=0.0015
p=0.0168
p<0.0001
p=0.0002
p=0.0406
p=0.0109
p=0.0007
p<0.0001
p<0.0001
p=0.0092
Control
Dox
TNFα
DOX+TNFα
LPS
DOX+LPS

Supplementary Fig. 9 | *Bcl11b* regulates genes involved in NF-kB signaling.

a, ChIP-seq tracks showing Bcl11b ChIP binding targets in “PI3K-AKT & mTOR signaling pathway”, “Wnt signaling pathway”, “Signaling pathways regulating pluripotency of stem cells” and “NFkappaB signaling pathway” The exon/intron diagram is shown at the bottom and arrowheads represent the direction of transcription.

b, Schematic diagram showing the putative regulation of NF-κB pathway by *Bcl11b*.

c, Real time PCR analysis of NF-κB pathway related gene expression after induction of *Bcl11b* expression. n=3 samples; bar and whiskers denote mean ± SD; two-tailed unpaired t-tests.

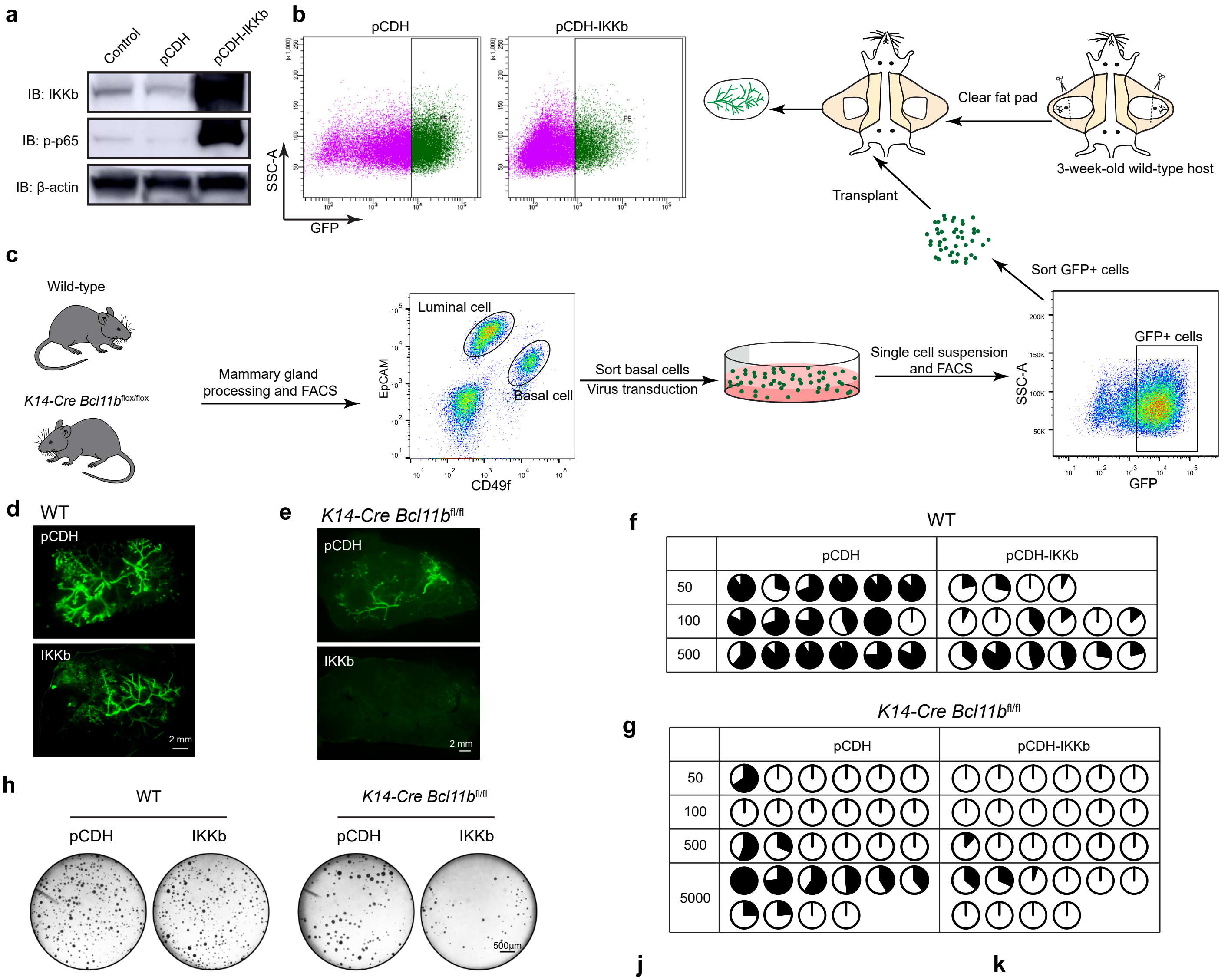

i

Limiting Dilution Transplant of WT and *K14-Cre Bcl11b*$^{fl/fl}$ Mammary Gland basal cells

	No. of Injected Cells/Fat Pad	Mammary Tree Outgrowth/No. of Injected Fat Pad	MRU frequency (95% Confidence Interval)
WT-pCDH	500	6 of 6	1 in 32 (1 in 15 to 69)
	100	5 of 6	
	50	6 of 6	
WT-IKKb	500	6 of 6	1 in 67 (1 in 31 to 148)
	100	4 of 6	
	50	3 of 4	
K14-Cre Bcl11b$^{fl/fl}$-pCDH	5000	8 of 10	1 in 2455 (1 in 1211 to 4977)
	500	2 of 6	
	100	0 of 6	
	50	1 of 6	
K14-Cre Bcl11b$^{fl/fl}$-IKKb	5000	3 of 10	1 in 11401 (1 in 4188 to 31036)
	500	1 of 6	
	100	0 of 6	
	50	0 of 6	

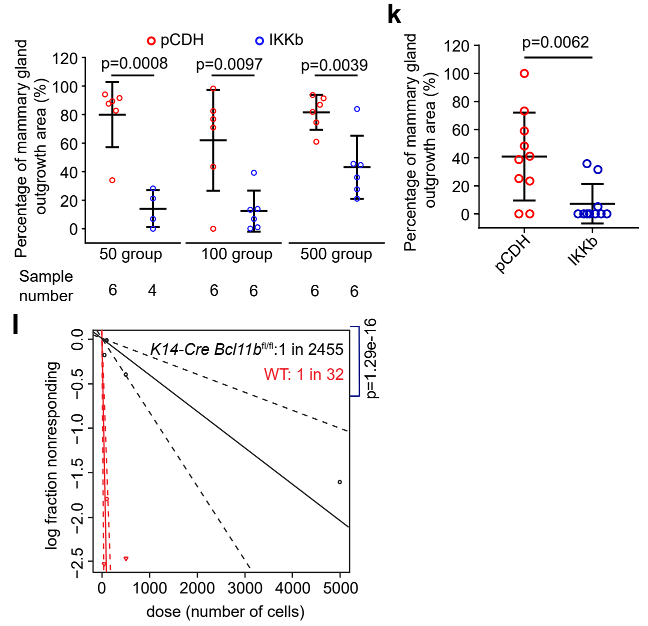

Supplementary Fig. 10 | NF-kB activation by IKKb overexpression drives stem cell exhaustion in the absence of *Bcl11b*.

a, Western blot analysis of IKKb and p-p65 expression in 293T cells upon enforced IKKb expression, n=3 samples.

b, FACS plot showing primary CD49f^{high}EpCAM^{low} cells transduced with pCDH or pCDH-IKKb vectors.

c, Experimental schematic diagram for limiting dilution transplants of WT and *K14-Cre Bcl11b*$^{fl/fl}$ CD49f^{high}EpCAM^{low} cells transduced with pCDH or pCDH-IKKb vectors.

d, Wholemount images of representative outgrowths of WT CD49f^{high}EpCAM^{low} cells transduced with pCDH (n=18 fat pad) or pCDH-IKKb (n=16 fat pad) vectors. Scale bar, 2 mm.

e, Wholemount images of representative outgrowths of *K14-Cre Bcl11b*$^{fl/fl}$ CD49f^{high}EpCAM^{low} cells transduced with pCDH (n=28 samples) or pCDH-IKKb (n=28 samples) vectors. Scale bar, 2 mm.

f, Pie chart of transplant of WT CD49f^{high}EpCAM^{low} cells transduced with pCDH or pCDH-IKKb vectors. n.s., not significant.

g, Pie chart of transplant of *K14-Cre Bcl11b*$^{fl/fl}$ CD49f^{high}EpCAM^{low} cells transduced with pCDH or pCDH-IKKb vectors. **P<0.01.

h, Representative colony formation assay images of CD49f ^{high}EpCAM^{low} cells from WT and *K14-Cre Bcl11b*$^{fl/fl}$ mice transduced with pCDH or pCDH-IKKb vectors. n=3 samples for each group, 3000 cells/well were seed and cultured for 1 week. Scale bar, 500μm.

i, Table for limiting dilution transplant of WT and *K14-Cre Bcl11b*$^{fl/fl}$ mammary gland CD49f^{high}EpCAM^{low} cells transduced with pCDH or pCDH-IKKb vectors.

j-k, Quantification of outgrowth area as proportion of total mammary gland area in WT (**j**) and *K14-Cre Bcl11b*$^{fl/fl}$ (**k,** n=10 samples for each group) groups. Statistical analysis was performed using two-tailed unpaired t-tests; bar and whiskers denote mean ± SD.

l, ELDA plot showing the comparison of WT and *K14-Cre Bcl11b*$^{fl/fl}$ CD49f^{high}EpCAM^{low} cells transduced with pCDH vectors.

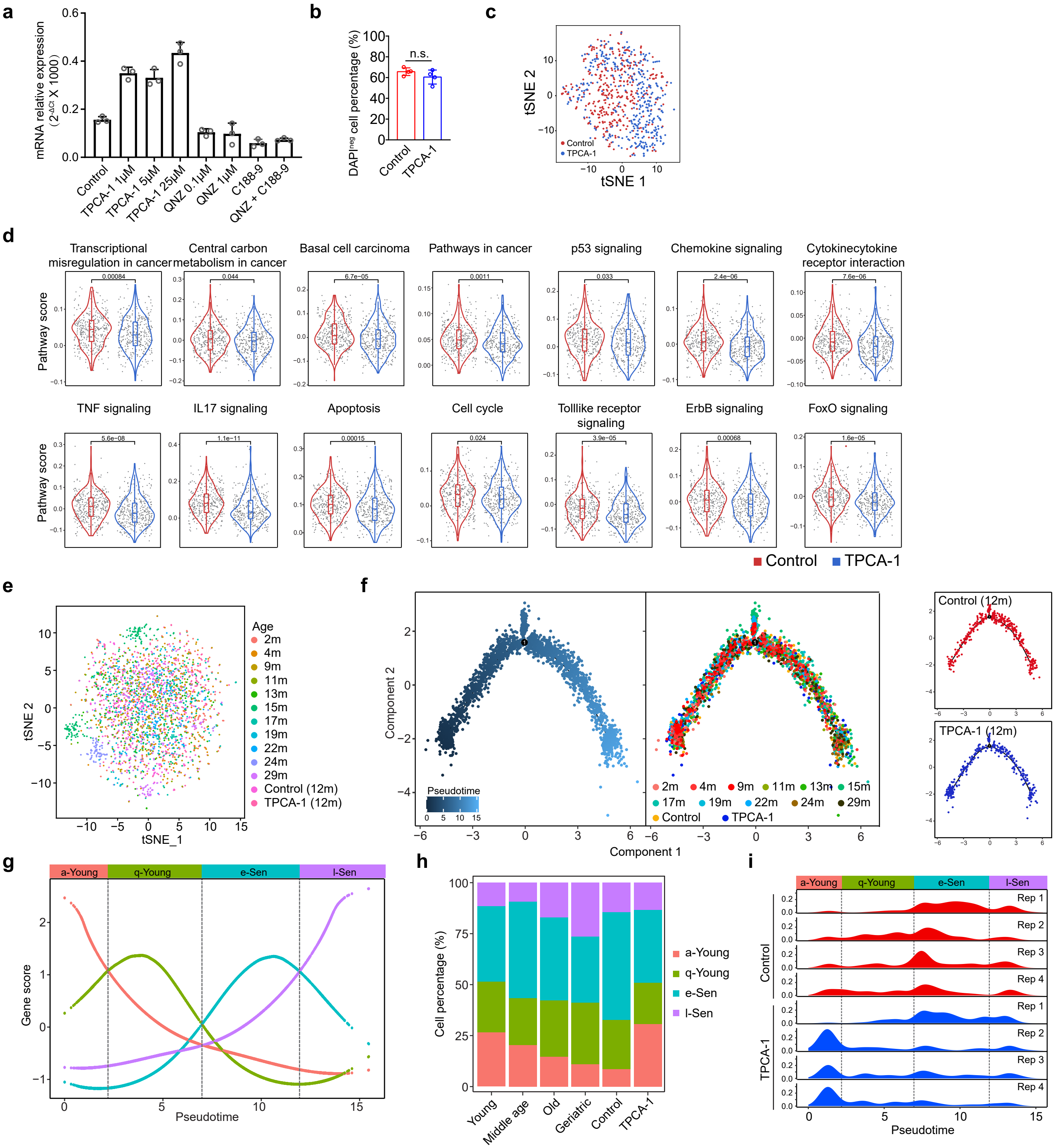
a
mRNA relative expression (2-ΔCt X 1000)
Control
TPCA-1 1μM
TPCA-1 5μM
TPCA-1 25μM
QNZ 0.1μM
QNZ 1μM
C188-9
QNZ + C188-9
b
DAPIneg cell percentage (%)
n.s.
Control
TPCA-1
c
tSNE 2
tSNE 1
Control
TPCA-1
d
Transcriptional misregulation in cancer
Central carbon metabolism in cancer
Basal cell carcinoma
Pathways in cancer
p53 signaling
Chemokine signaling
Cytokinecytokine receptor interaction
Pathway score
0.00084
0.044
6.7e−05
0.0011
0.033
2.4e−06
7.6e−06
TNF signaling
IL17 signaling
Apoptosis
Cell cycle
Tolllike receptor signaling
ErbB signaling
FoxO signaling
5.6e−08
1.1e−11
0.00015
0.024
3.9e−05
0.00068
1.6e−05
Control
TPCA-1
e
Age
2m
4m
9m
11m
13m
15m
17m
19m
22m
24m
29m
Control (12m)
TPCA-1 (12m)
tSNE 2
tSNE_1
f
Component 2
Component 1
Pseudotime
Control (12m)
TPCA-1 (12m)
Control
TPCA-1
g
a-Young
q-Young
e-Sen
l-Sen
Gene score
Pseudotime
h
Cell percentage (%)
Young
Middle age
Old
Geriatric
Control
TPCA-1
a-Young
q-Young
e-Sen
l-Sen
i
a-Young
q-Young
e-Sen
l-Sen
Control
TPCA-1
Rep 1
Rep 2
Rep 3
Rep 4
Pseudotime

Supplementary Fig. 11 | TPCA-1 reshapes ageing programs at the transcriptome level.

a, Real time PCR quantification of *Bcl11b* expression levels treated with QNZ, C188-9 and TPCA-1 with β-actin as an internal control (n=3 samples) in Comma Dβ cells.

b, Percentage of DAPI negative cells in primary basal cells treated with TPCA-1. n.s., not significant.

c, tSNE plot showing the clustering of $CD49f^{high}EpCAM^{low}$ cells from 12m-old month WT mice with (367 cells from 4 mice) or without (373 cells from 4 mice) TPCA-1 treatment.

d, Down-regulated signaling pathways upon TPCA-1 treatment. Two-sided wilcoxon -test was used.

e, tSNE plot showing the clustering of $CD49f^{high}EpCAM^{low}$ cells from different age WT mice and 12m-old month WT mice with or without TPCA-1 treatment.

f, Pseudotime-ordering analysis of single cell transcriptomes from WT mice of various ages and 12m-old month WT mice with or without TPCA-1 treatment. Cell numbers are listed in the Supplementary Data 1.

g, Cell state signature gene scores over the pseudotime.

h, Cell proportion of 4 state cells in young, middle age, old, geriatric, control (12month) and TPCA-1 (12 month) groups. Cell numbers are listed in the Supplementary Data 1.

i, Cell density distribution of $CD49f^{high}EpCAM^{low}$ cells in control (12month) and TPCA-1 (12month) groups along with pseudotime.

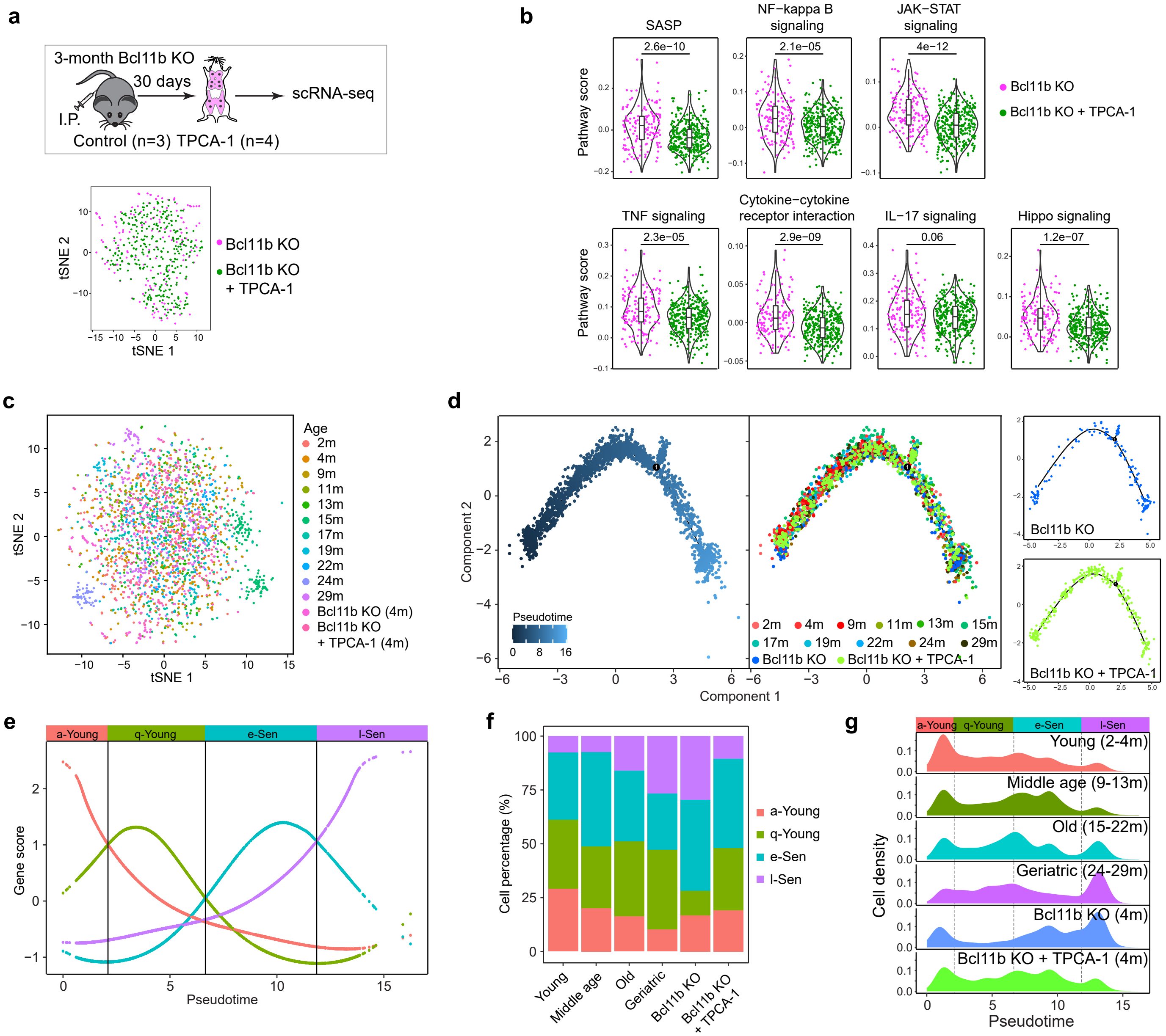
a
3-month Bcl11b KO
30 days
scRNA-seq
I.P.
Control (n=3) TPCA-1 (n=4)
tSNE 2
tSNE 1
Bcl11b KO
Bcl11b KO + TPCA-1
b
SASP
NF−kappa B signaling
JAK−STAT signaling
Pathway score
2.6e−10
2.1e−05
4e−12
Bcl11b KO
Bcl11b KO + TPCA-1
TNF signaling
Cytokine−cytokine receptor interaction
IL−17 signaling
Hippo signaling
2.3e−05
2.9e−09
0.06
1.2e−07
c
Age
2m
4m
9m
11m
13m
15m
17m
19m
22m
24m
29m
Bcl11b KO (4m)
Bcl11b KO + TPCA-1 (4m)
d
Component 2
Component 1
Pseudotime
Bcl11b KO
Bcl11b KO + TPCA-1
e
a-Young
q-Young
e-Sen
l-Sen
Gene score
Pseudotime
f
Cell percentage (%)
Young
Middle age
Old
Geriatric
Bcl11b KO
Bcl11b KO + TPCA-1
g
Young (2-4m)
Middle age (9-13m)
Old (15-22m)
Geriatric (24-29m)
Bcl11b KO (4m)
Bcl11b KO + TPCA-1 (4m)
Cell density
Pseudotime

Supplementary Fig. 12 | TPCA-1 can rescue Bcl11b KO derived ageing clock.

a, Schematic diagram showing the strategy of TPCA-1 (10mg/kg) treatment in vivo on 3-month-old *K14-Cre Bcl11b*$^{\mathrm{fl/fl}}$ mice. tSNE plot showing the clustering of CD49f^{high}EpCAM^{low} cells from 4-month-old *K14-Cre Bcl11b*$^{\mathrm{fl/fl}}$ mice with (341 cells from 4 mice) or without (149 cells from 3 mice) TPCA-1 treatment.

b, Down-regulated signaling pathways upon TPCA-1 treatment.

c, tSNE plot showing the clustering of CD49f^{high}EpCAM^{low} cells from different age WT mice and 4-month-old *K14-Cre Bcl11b*$^{\mathrm{fl/fl}}$ mice with or without TPCA-1 treatment.

d, Pseudotime-ordering analysis of single cell transcriptomes from WT mice of various ages and 4-month-old *K14-Cre Bcl11b*$^{\mathrm{fl/fl}}$ mice with (341 cells from 4 mice) or without (149 cells from 3 mice) TPCA-1 treatment.

e, Cell state signature gene scores over the pseudotime.

f, Cell proportion of 4 state cells in young, middle age, old, geriatric, control (4 month) and TPCA-1 (4 month) groups.

g, Cell density distribution of CD49f^{high}EpCAM^{low} cells in control (4 month) and TPCA-1 (4 month) groups along with pseudotime.

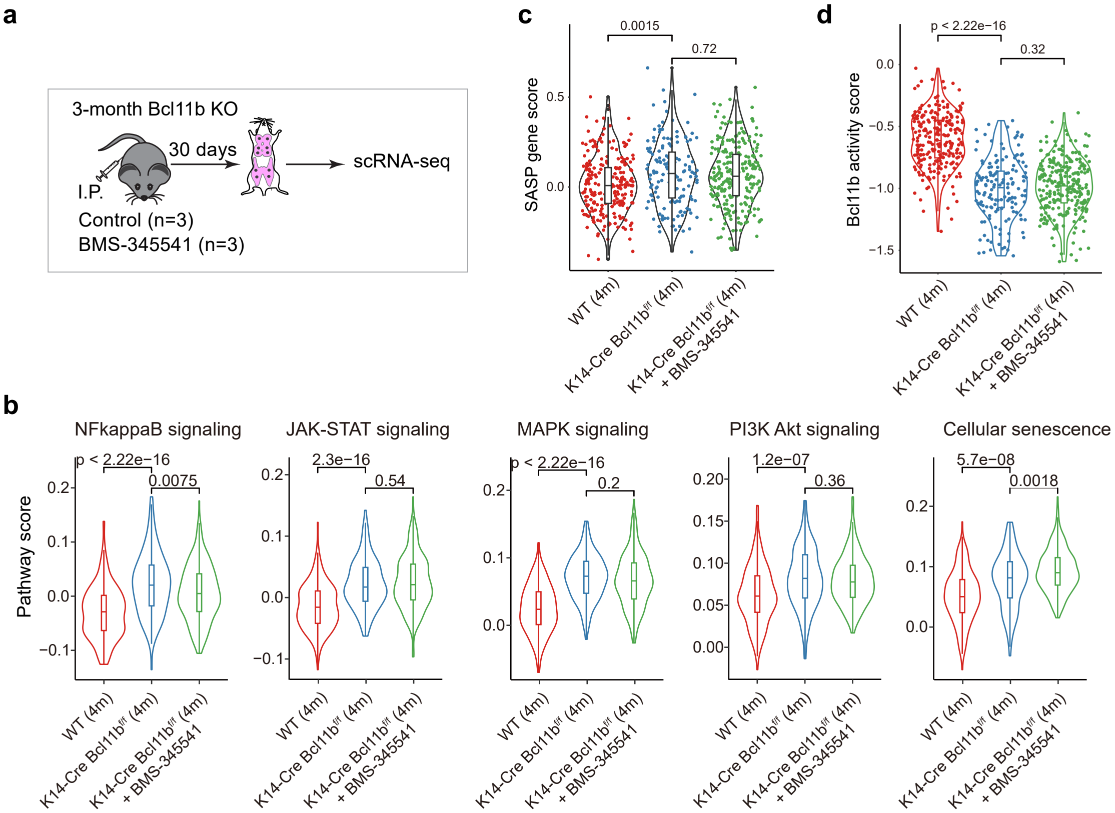
a
3-month Bcl11b KO
30 days
I.P.
scRNA-seq
Control (n=3)
BMS-345541 (n=3)
b
NFkappaB signaling
JAK-STAT signaling
MAPK signaling
PI3K Akt signaling
Cellular senescence
Pathway score
p < 2.22e−16
0.0075
2.3e−16
0.54
p < 2.22e−16
0.2
1.2e−07
0.36
5.7e−08
0.0018
c
SASP gene score
0.0015
0.72
d
Bcl11b activity score
p < 2.22e−16
0.32
WT (4m)
K14-Cre Bcl11b$^{f/f}$ (4m)
K14-Cre Bcl11b$^{f/f}$ (4m) + BMS-345541

Supplementary Fig. 13 | Bcl11b KO derived ageing clock can't be rescued by the BMS-345541 treatment.

a, Schematic diagram showing the strategy of BMS-345541 (35mg/kg) treatment in vivo on 3-month-old *K14-Cre Bcl11b*$^{fl/fl}$ mice. 4 months old WT(236 cells from 3 mice), *K14-Cre Bcl11b*$^{fl/fl}$ (149 cells from 3mice) and BMS-345541 (256 cells from 3 mice) treated *K14-Cre Bcl11b*$^{fl/fl}$ mice were used. Two-sided wilcoxon -test was used.

b, Aging-regulated signaling pathway score upon BMS-345541 treatment.

c, Senescence related pathway score in CD49f^{high}EpCAM^{low} cells from WT mice and 4-month-old *K14-Cre Bcl11b*$^{fl/fl}$ mice with or without BMS-345541 treatment.

d, Violin plots showing the *Bcl11b* activity score in CD49f^{high}EpCAM^{low} cells from WT mice and 4-month-old *K14-Cre Bcl11b*$^{fl/fl}$ mice with or without BMS-345541 treatment.

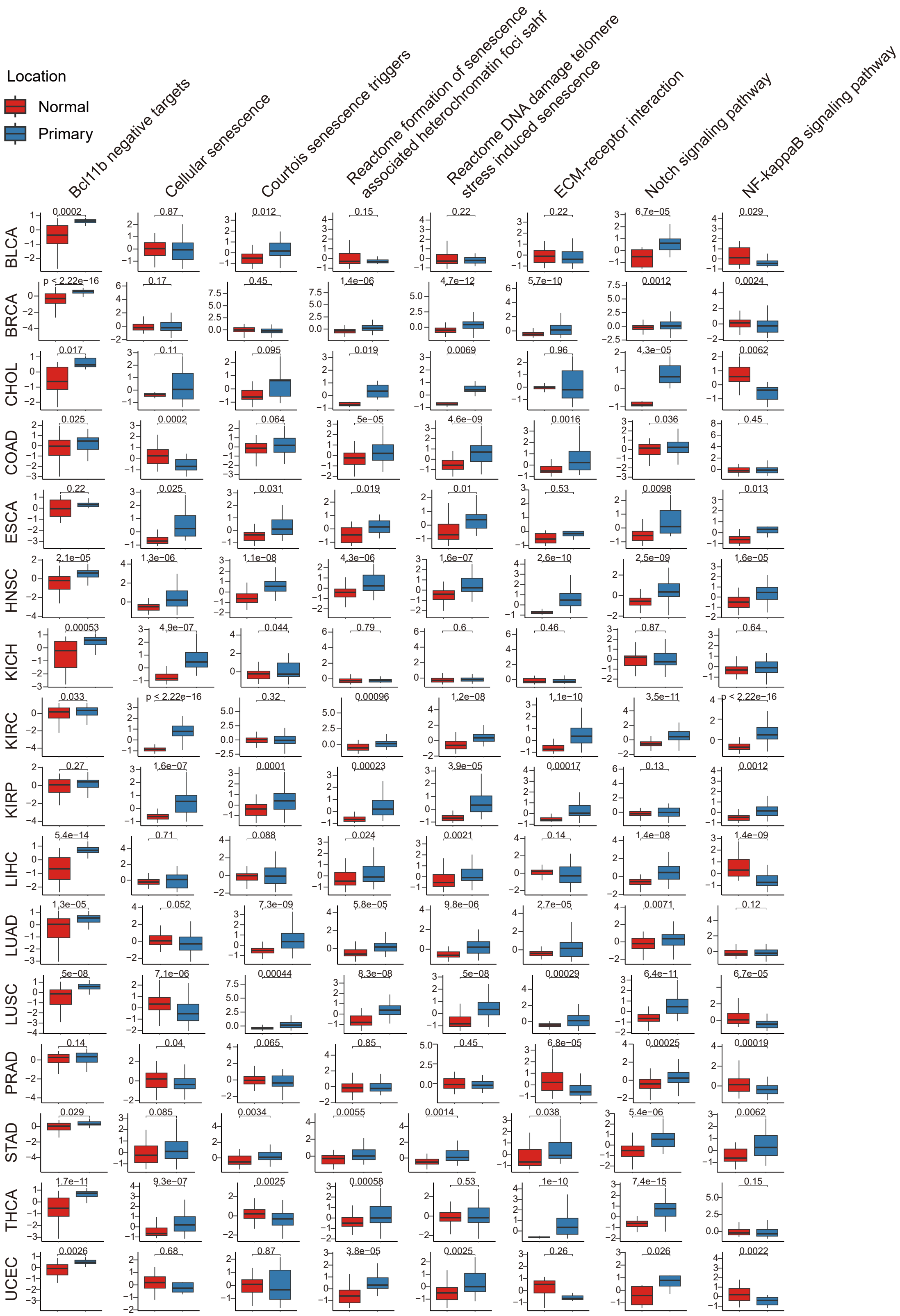
Location
Normal
Primary
Bcl11b negative targets
Cellular senescence
Courtois senescence triggers
Reactome formation of senescence associated heterochromatin foci sahf
Reactome DNA damage telomere stress induced senescence
ECM-receptor interaction
Notch signaling pathway
NF-kappaB signaling pathway
BLCA 0.0002 0.87 0.012 0.15 0.22 0.22 6.7e-05 0.029
BRCA p < 2.22e-16 0.17 0.45 1.4e-06 4.7e-12 5.7e-10 0.0012 0.0024
CHOL 0.017 0.11 0.095 0.019 0.0069 0.96 4.3e-05 0.0062
COAD 0.025 0.0002 0.064 5e-05 4.6e-09 0.0016 0.036 0.45
ESCA 0.22 0.025 0.031 0.019 0.01 0.53 0.0098 0.013
HNSC 2.1e-05 1.3e-06 1.1e-08 4.3e-06 1.6e-07 2.6e-10 2.5e-09 1.6e-05
KICH 0.00053 4.9e-07 0.044 0.79 0.6 0.46 0.87 0.64
KIRC 0.033 p < 2.22e-16 0.32 0.00096 1.2e-08 1.1e-10 3.5e-11 p < 2.22e-16
KIRP 0.27 1.6e-07 0.0001 0.00023 3.9e-05 0.00017 0.13 0.0012
LIHC 5.4e-14 0.71 0.088 0.024 0.0021 0.14 1.4e-08 1.4e-09
LUAD 1.3e-05 0.052 7.3e-09 5.8e-05 9.8e-06 2.7e-05 0.0071 0.12
LUSC 5e-08 7.1e-06 0.00044 8.3e-08 5e-08 0.00029 6.4e-11 6.7e-05
PRAD 0.14 0.04 0.065 0.85 0.45 6.8e-05 0.00025 0.00019
STAD 0.029 0.085 0.0034 0.0055 0.0014 0.038 5.4e-06 0.0062
THCA 1.7e-11 9.3e-07 0.0025 0.00058 0.53 1e-10 7.4e-15 0.15
UCEC 0.0026 0.68 0.87 3.8e-05 0.0025 0.26 0.026 0.0022

Supplementary Fig. 14 | Pathway and activity analysis across various cancer types.

Bcl11b's activity and aging-regulated signaling pathway score in normal and primary cancer tissues. Paired tumor adjacent tissue sample and primary cancer sample from TCGA were used, including 19 patients with BLCA, 112 patients with BRCA, 9 patients with CHOL, 49 patients with COAD, 11 patients with ESCA, 43 patients with HNSC, 25 patients with KICH, 71 patients with KIRC, 32 patients with KIRP, 50 patients with LIHC, 58 patients with LUAD, 50 patients with LUSC, 52 patients with PRAD, 32 patients with STAD, 58 patients with THCA and 22 patients with UCEC. Two-tailed paired t-test was used.
