# Supplementary material for: Progressive senescence programs induce intrinsic vulnerability to aging-related female breast cancer
Source: Nat Commun. 2024 Jun 17;15:5154. doi: 10.1038/s41467-024-49106-2 (PMC11183265; doi:10.1038/s41467-024-49106-2)
Supplement: Supplementary file 2 — Description of Additional Supplementary Files [file 41467_2024_49106_MOESM2_ESM.pdf]

Description of Additional Supplementary Files

Supplemental Data 1. Single cell RNA-seq sample information.

Supplemental Data 2. Transcription factors enriched in diffrent cell states.

Supplemental Data 3. Bcl11b binding targets identified by ChIP-seq.

Supplemental Data 4. Pathways enriched in wt and Bcl11b ko cells.

Supplemental Data 5. KEGG and GO pathway enrichment analysis revealed that many of the aging-associated pathways were the direct targets.

Supplemental Data 6. Up-regulated and Down-regulated signaling pathways upon TPCA-1 treatment.
