# Supplementary material for: Progressive senescence programs induce intrinsic vulnerability to aging-related female breast cancer
Source: Nat Commun. 2024 Jun 17;15:5154. doi: 10.1038/s41467-024-49106-2 (PMC11183265; doi:10.1038/s41467-024-49106-2)
Supplement: Supplementary file 9 — Reporting Summary [file 41467_2024_49106_MOESM9_ESM.pdf]

nature portfolio

Corresponding author(s): Shang Cai

Last updated by author(s): Apr 2, 2024

Reporting Summary

Nature Portfolio wishes to improve the reproducibility of the work that we publish. This form provides structure for consistency and transparency in reporting. For further information on Nature Portfolio policies, see our Editorial Policies and the Editorial Policy Checklist.

Statistics

For all statistical analyses, confirm that the following items are present in the figure legend, table legend, main text, or Methods section.

n/a	Confirmed	
☐	☒	The exact sample size (*n*) for each experimental group/condition, given as a discrete number and unit of measurement
☐	☒	A statement on whether measurements were taken from distinct samples or whether the same sample was measured repeatedly
☐	☒	The statistical test(s) used AND whether they are one- or two-sided<br>*Only common tests should be described solely by name; describe more complex techniques in the Methods section.*
☒	☐	A description of all covariates tested
☒	☐	A description of any assumptions or corrections, such as tests of normality and adjustment for multiple comparisons
☐	☒	A full description of the statistical parameters including central tendency (e.g. means) or other basic estimates (e.g. regression coefficient) AND variation (e.g. standard deviation) or associated estimates of uncertainty (e.g. confidence intervals)
☐	☒	For null hypothesis testing, the test statistic (e.g. *F*, *t*, *r*) with confidence intervals, effect sizes, degrees of freedom and *P* value noted<br>*Give P values as exact values whenever suitable.*
☒	☐	For Bayesian analysis, information on the choice of priors and Markov chain Monte Carlo settings
☒	☐	For hierarchical and complex designs, identification of the appropriate level for tests and full reporting of outcomes
☒	☐	Estimates of effect sizes (e.g. Cohen's *d*, Pearson's *r*), indicating how they were calculated

Our web collection on statistics for biologists contains articles on many of the points above.

Software and code

Policy information about availability of computer code

Data collection	Whole mount images were obtained using a stereomicroscope (Nikon, SMZ18).<br>Western blot imaged by Gel imaging system (GE, AI680RGB)<br>Luciferase activity results were tested using the microplate reader (Thermo, Varioskan LUX).<br>Flow cytometrhy files were collected using FACS Aria II (BD Bioscience).<br>Immunohistochemistry and HE staining Images were obtained using Eclipse Ti2 inverted microscope (Nikon).<br>scRNA-sequencing results were collected using Illumina novaseq 6000 platform (Novogene).
Data analysis	All custom code and scripts used in this study are provided in supplementary data of this manuscript. Other software packages include: GraphPad Prism 7.00, Image J, FlowJo (Ver 10), PROSize 3.0, ELDA (http://bioinf.wehi.edu.au/software/elda/), trim_galore(Ver.0.6.7), fastqc (Ver.0.11.9), umi_tools(Ver.1.1.1), subread(Ver.2.0.2), STAR(Ver.2.7.8), samtools(Ver.1.13), Seurat(Ver 4.2.0)<br>monocle(Ver 2.24.1), reshape2(Ver 1.4.4), ggplot2(Ver 3.5.0), ComplexHeatmap(Ver 2.12.1), clusterProfiler(Ver 4.7.1.002), org.Mm.eg.db(Ver 3.15.0), ChIPseeker(Ver 1.32.1), GenomicFeatures(Ver 1.48.3), TxDb.Mmusculus.UCSC.mm10.knownGene(Ver 3.10.0)

For manuscripts utilizing custom algorithms or software that are central to the research but not yet described in published literature, software must be made available to editors and reviewers. We strongly encourage code deposition in a community repository (e.g. GitHub). See the Nature Portfolio guidelines for submitting code & software for further information.

Data

Policy information about availability of data

All manuscripts must include a data availability statement. This statement should provide the following information, where applicable:

- Accession codes, unique identifiers, or web links for publicly available datasets
- A description of any restrictions on data availability
- For clinical datasets or third party data, please ensure that the statement adheres to our policy

All data relevant to this study are available from the corresponding authors upon reasonable request. scRNA-seq data were deposited in GEO database under the accession number GSE195647.To review GEO accession GSE195647: Go to https://www.ncbi.nlm.nih.gov/geo/query/acc.cgi?acc=GSE195647. Enter token wxmhkmaqvtmxpqr into the box. TCGA data is from https://www.cancer.gov/ccg/research/genome-sequencing/tcga. The datasets used to perform transcriptor factor enrichment analysis are from the following site: ENCODE, https://maayanlab.cloud/Harmonizome/dataset/ENCODE+Transcription+Factor+Binding+Site+Profiles; TRRUST v2, https://www.grnpedia.org/trrust; CHEA, https://maayanlab.cloud/chea3/. The mouse genome mm10(M25) was obtained from GENCODE database.

Human research participants

Policy information about studies involving human research participants and Sex and Gender in Research.

Reporting on sex and gender	N/A
Population characteristics	N/A
Recruitment	N/A
Ethics oversight	N/A

Note that full information on the approval of the study protocol must also be provided in the manuscript.

Field-specific reporting

Please select the one below that is the best fit for your research. If you are not sure, read the appropriate sections before making your selection.

☒ Life sciences ☐ Behavioural & social sciences ☐ Ecological, evolutionary & environmental sciences

For a reference copy of the document with all sections, see nature.com/documents/nr-reporting-summary-flat.pdf

Life sciences study design

All studies must disclose on these points even when the disclosure is negative.

Sample size	We used 25 mice to do the scRNA-seq to make sure we could build the aging trajectory. We used more than 3 mice to test the drug effect.
Data exclusions	Low quality scRNA-seq data were excluded.
Replication	Each result described in the paper is based on at least two independent biological replicates for animal experiments. Further details are described in each figure legend.
Randomization	Mice studies: Age and background-matched mice were randomly allocated into the groups for all animal experiments.
Blinding	We didn't perform blinding for in vivo measurement, as blinding was considered not to affect the measurement result. Other analysis were performed without informing the investigators with the group allocation.

Reporting for specific materials, systems and methods

We require information from authors about some types of materials, experimental systems and methods used in many studies. Here, indicate whether each material, system or method listed is relevant to your study. If you are not sure if a list item applies to your research, read the appropriate section before selecting a response.

Materials & experimental systems

n/a	Involved in the study
[ ]	[x] Antibodies
[ ]	[x] Eukaryotic cell lines
[x]	[ ] Palaeontology and archaeology
[ ]	[x] Animals and other organisms
[x]	[ ] Clinical data
[x]	[ ] Dual use research of concern

Methods

n/a	Involved in the study
[ ]	[x] ChIP-seq
[ ]	[x] Flow cytometry
[x]	[ ] MRI-based neuroimaging

Antibodies

Antibodies used

-V450 Rat Anti-Mouse CD45 (1:200; 560501;BD)
-BV421 Rat Anti-Mouse CD31 (1:200; 562939;BD)
-V450 Rat Anti-Mouse TER-119/Erythroid Cells (1:200; 560504;BD)
-FITC anti-human/mouse CD49f Antibody (1:200; 313606; BioLegend)
-PerCP/Cyanine5.5 anti-mouse CD326 (Ep-CAM) Antibody (1:200; 118220; BioLegend)
-Rat anti-Bcl11b (1:1000; ab18465; abcam)
-Rabbit anti-IKKβ (1:1000; 2370; Cell signaling technology)
-Rabbit anti-p-IKKα/β (1:1000; 2697; Cell signaling technology)
-Rabbit anti-p-p65 (1:1000; 3033; Cell signaling technology)
-Rabbit anti-p65 (1:1000; 8242; Cell signaling technology)
-Mouse anti-IκBα (1:1000; 9247; Cell signaling technology)
-HRP-Donkey anti mouse (1:10000; 7076S; Cell signaling technology)
-HRP-Donkey anti rat (1:10000; 7077S; Cell signaling technology)
-HRP-Donkey anti rabbit (1:10000; 7074S; Cell signaling technology)
-Rabbit anti-p-p65 (1:50; ab131100; Abcam)
-Rabbit anti-Il-6 (1:100; NB600-1131; NOVUS)
-Mouse anti-Ssea1 (1:200; ab16285; Abcam)
-Rabbit anti-Oct4 (1:200; ab19857; Abcam)
-Rabbit anti-IgG (ab172730; Abcam)
-Rabbit anti-Bcl11b (A300-384A; Benthyl laboratories.inc)

Validation

-V450 Rat Anti-Mouse CD45 (1:200; 560501;BD)
https://www.bdbiosciences.com/en-eu/products/reagents/flow-cytometry-reagents/research-reagents/single-color-antibodies-ruo/v450-rat-anti-mouse-cd45.560501
-BV421 Rat Anti-Mouse CD31 (1:200; 562939;BD)
https://www.bdbiosciences.com/en-eu/products/reagents/flow-cytometry-reagents/research-reagents/single-color-antibodies-ruo/bv421-rat-anti-mouse-cd31.562939
-V450 Rat Anti-Mouse TER-119/Erythroid Cells (1:200; 560504;BD)
https://www.bdbiosciences.com/en-eu/products/reagents/flow-cytometry-reagents/research-reagents/single-color-antibodies-ruo/v450-rat-anti-mouse-ter-119-erythroid-cells.560504
-FITC anti-human/mouse CD49f Antibody (1:200; 313606; BioLegend)
https://www.biolegend.com/en-us/products/fitc-anti-human-mouse-cd49f-antibody-2606
-PerCP/Cyanine5.5 anti-mouse CD326 (Ep-CAM) Antibody (1:200; 118220; BioLegend)
https://www.biolegend.com/en-us/products/percp-cyanine5-5-anti-mouse-cd326-ep-cam-antibody-5602
-Rat anti-Bcl11b (1:1000; ab18465; abcam)
https://www.abcam.com/ctip2-antibody-25b6-ab18465.html
-Rabbit anti-IKKβ (1:1000; 2370; Cell signaling technology)
https://www.cellsignal.cn/products/primary-antibodies/ikkb-2c8-rabbit-mab/2370
-Rabbit anti-p-IKKα/β (1:1000; 2697; Cell signaling technology)
https://www.cellsignal.cn/products/primary-antibodies/phospho-ikka-b-ser176-180-16a6-rabbit-mab/2697?site-search-type=Products&N=4294956287&Ntt=2697&fromPage=plp&_requestid=915796
-Rabbit anti-p-p65 (1:1000; 3033; Cell signaling technology)
https://www.cellsignal.cn/products/primary-antibodies/phospho-nf-kb-p65-ser536-93h1-rabbit-mab/3033?site-search-type=Products&N=4294956287&Ntt=3033&fromPage=plp&_requestid=916203
-Rabbit anti-p65 (1:1000; 8242; Cell signaling technology)
https://www.cellsignal.cn/products/primary-antibodies/nf-kb-p65-d14e12-xp-rabbit-mab/8242?site-search-type=Products&N=4294956287&Ntt=8242&fromPage=plp&_requestid=916250
-Mouse anti-IκBα (1:1000; 9247; Cell signaling technology)
https://www.cellsignal.cn/products/primary-antibodies/ikba-112b2-mouse-mab-carboxy-terminal-antigen/9247?site-search-type=Products&N=4294956287&Ntt=9247&fromPage=plp&_requestid=916371
-HRP-Donkey anti mouse (1:10000; 7076S; Cell signaling technology)
https://www.cellsignal.cn/products/secondary-antibodies/anti-mouse-igg-hrp-linked-antibody/7076?site-search-type=Products&N=4294956287&Ntt=7076s&fromPage=plp&_requestid=916446
-HRP-Donkey anti rat (1:10000; 7077S; Cell signaling technology)

https://www.cellsignal.cn/products/secondary-antibodies/anti-rat-igg-hrp-linked-antibody/7077?site-search-type=Products&N=4294956287&Ntt=7077&fromPage=plp&_requestid=916501
-HRP-Donkey anti rabbit (1:10000; 7074S; Cell signaling technology)
https://www.cellsignal.cn/products/secondary-antibodies/anti-rabbit-igg-hrp-linked-antibody/7074?site-search-type=Products&N=4294956287&Ntt=7074&fromPage=plp&_requestid=916554
-Rabbit anti-p-p65 (1:50; ab131100; Abcam)
https://www.abcam.com/nf-kb-p65-phospho-t254-antibody-ab131100.html
-Rabbit anti-Il-6 (1:100; NB600-1131; NOVUS)
https://www.novusbio.com/products/il-6-antibody_nb600-1131
-Mouse anti-Ssea1 (1:200; ab16285; Abcam)
https://www.abcam.com/ssea1-antibody-mc-480-ab16285.html
-Rabbit anti-Oct4 (1:200; ab19857; Abcam)
https://www.abcam.com/oct4-antibody-ab19857.html
-Rabbit anti-IgG (ab172730; Abcam)
https://www.abcam.com/rabbit-igg-monoclonal-epr25a-isotype-control-ab172730.html
-Rabbit anti-Bcl11b (A300-384A; Benthyl laboratories.inc)
https://www.citeab.com/antibodies/654910-a300-372a-rabbit-anti-pbef-antibody-affinity-purifie

Eukaryotic cell lines

Policy information about cell lines and Sex and Gender in Research

Cell line source(s)	Comma D beta cell line was kindly provided by Dr. Medina.
Authentication	All cell lines were not authenticated.
Mycoplasma contamination	All cell lines used in our study were negative for mycoplasma contamination.
Commonly misidentified lines (See ICLAC register)	No commonly misidentified cell line was used.

Animals and other research organisms

Policy information about studies involving animals; ARRIVE guidelines recommended for reporting animal research, and Sex and Gender in Research

Laboratory animals	The 2-4 month Bcl11bflox/flox mice (C57BL/6 background) were generously provided by Mark Leid's lab and the 2-4 month B6N.Cg-Tg (KRT14-cre)1Amc/J (stock number 018964) were purchased from Jackson Laboratory. 2-4 month mTmG mice (B6.129(Cg)-Gt (ROSA)26Sortm4(ACTB-tdTomato,-EGFP)Luo/J, 007676) were purchased from The Jackson Laboratory (The Jackson Laboratory, Bar Harbor, Maine, USA). Female C57BL/6 mice, 2-6 months old were purchased from Jackson Laboratory, and were maintained till 29 months.
Wild animals	This project did not use wild animals.
Reporting on sex	This study only applied to the mammary glands of female mice.
Field-collected samples	This project did not involve samples collected from the field.
Ethics oversight	Animals were housed in a specific pathogen-free conditions and fed standard mouse chow. All animal experiments were carried out in compliance with China laws and regulations. The local institutional animal ethics board (Institutional Animal Care and Use Committee of Westlake University) approved all mouse experiments (permission numbers: 19-001-2-CS). Experiments were performed in accordance with government and institutional guidelines and regulations. All mice are housed at 20–24 °C with 40–60% humidity, and 12-h cycle of light/darkness (7 a.m. to 7 p.m.).

Note that full information on the approval of the study protocol must also be provided in the manuscript.

ChIP-seq

Data deposition

- [x] Confirm that both raw and final processed data have been deposited in a public database such as GEO.
- [x] Confirm that you have deposited or provided access to graph files (e.g. BED files) for the called peaks.

Data access links *May remain private before publication.*	We have already deposited the ChIP-seq data to GEO database under the accession number GSE195647. To review GEO accession GSE195647: Go to https://www.ncbi.nlm.nih.gov/geo/query/acc.cgi?acc=GSE195647, enter token wxmhkmaqvtmxpqr into the box.
Files in database submission	GSM5842356 bclrb12 ChIP-seq data<br>GSM5842357 IgG12 ChIP-seq data

	GSE195647_bclrb12_IgG12_peaks.narrowPeak.gz peak file
Genome browser session (e.g. UCSC)	http://genome.ucsc.edu/s/liuxiaoqin/bcl11b%2Dliuxiaoqin

Methodology

Replicates	There is no replicate for Bcl11b ChIP-seq experiment.
Sequencing depth	We used the pair-end 150 sequencing technology . For anti-Bcl11b group, we got 22423689 reads, in which 12135526 were uniquely mapped to mm10 genome. For IgG group, we got 24037632 reads, in which 10109748 were uniquely mapped to mm10 genome.
Antibodies	-Rabbit anti-IgG (ab172730; Abcam)<br>-Rabbit anti-Bcl11b (A300-384A; Benthyl laboratories.inc)
Peak calling parameters	TrimGalore trimmed reads were mapped to mm10 with bowtie2 (default parameter). Duplicated reads were marked with MarkDuplicates in picardtools and filtered out with samtools (-F 1804 -f 2 -q 30). For peak calling, we used MACS2 with the following parameter: -f BAMPE -g mm --keep-dup all --nomodel.
Data quality	We got 1197 peaks for Bcl11b binding site, among which 1060 peaks were at FDR 5% and above 5-fold enrichment.
Software	trim_galore(Ver.0.6.7)<br>bowtie2(Ver.2.4.2)<br>picardtools(Ver.1.119)<br>MACS2(Ver.2.2.7.1)

Flow Cytometry

Plots

Confirm that:

- [x] The axis labels state the marker and fluorochrome used (e.g. CD4-FITC).
- [x] The axis scales are clearly visible. Include numbers along axes only for bottom left plot of group (a 'group' is an analysis of identical markers).
- [x] All plots are contour plots with outliers or pseudocolor plots.
- [x] A numerical value for number of cells or percentage (with statistics) is provided.

Methodology

Sample preparation	Mammary glands were collected from 2nd, 3rd, 4th pair of mammary glands of C57/BL6 mice, and were dissected and processed according to the published protocol 80 with minor revision. Mammary glands were minced into 1 mm3 size using a tissue cutting blade and digested with 0.5 mg/mL Collagenase type III (Worthington, LS004182) and 50 U/mL hyaluronidase (Worthington, LS002592) for 2 hours with gentle pipetting every 30 mins. Digested mammary homogenate was collected and treated with ACK lysing buffer for 5 mins on ice, then was digested using 0.25% Trypsin-EDTA (GIBCO) for 5 mins, followed by DNase I (Worthington, LS002139) digestion. After filtered by 70 μm strainer, the dissociated mammary cells were stained with CD45 (Biolegend), CD31 (Biolegend), Ter119 (Biolegend), EpCAM (Biolegend), CD49f (Biolegend) for 20 mins on ice. Cells were washed and resuspended in HBSS+2% FBS+1% PSA+DAPI (1 μg/mL), then were sorted using FACS Aria II (BD Bioscience).
Instrument	BD FACAria Fusion SORP; Beckman Coulter MoFlo
Software	FlowJo_v10
Cell population abundance	We targeted CD49f-FITC high EpCAM-PerCP cy5.5 middle Lineage negative population.
Gating strategy	FSC/SSC gate for elimination of debris followed by a gate for DAPI- CD45-CD31-Ter119-. Then we gate CD49f-FITC high EpCAM-PerCP cy5.5 middle population.

- [x] Tick this box to confirm that a figure exemplifying the gating strategy is provided in the Supplementary Information.
