# Supplementary material for: Progressive senescence programs induce intrinsic vulnerability to aging-related female breast cancer
Source: Nat Commun. 2024 Jun 17;15:5154. doi: 10.1038/s41467-024-49106-2 (PMC11183265; doi:10.1038/s41467-024-49106-2)
Supplement: Supplementary file 10 — Peer Review [file 41467_2024_49106_MOESM10_ESM.pdf]

nature portfolio

Peer Review File

Progressive Senescence Programs Induce Intrinsic Vulnerability to Aging-related Female Breast Cancer

Open Access This file is licensed under a Creative Commons Attribution 4.0 International License, which permits use, sharing, adaptation, distribution and reproduction in any medium or format, as long as you give appropriate credit to the original author(s) and the source, provide a link to the Creative Commons license, and indicate if changes were made. In the cases where the authors are anonymous, such as is the case for the reports of anonymous peer reviewers, author attribution should be to 'Anonymous Referee' followed by a clear attribution to the source work. The images or other third party material in this file are included in the article's Creative Commons license, unless indicated otherwise in a credit line to the material. If material is not included in the article's Creative Commons license and your intended use is not permitted by statutory regulation or exceeds the permitted use, you will need to obtain permission directly from the copyright holder. To view a copy of this license, visit http://creativecommons.org/licenses/by/4.0/.

Editorial Note: Parts of this Peer Review File have been redacted as indicated to remove third-party material where no permission to publish could be obtained.

REVIEWER COMMENTS

Reviewer #1 (Remarks to the Author):

The study by Bai and colleagues aimed to understand the mechanism underlying increased cancer incidence during aging using mammary gland as the primary working model. They characterized the aging-associated mammary basal stem/progenitor cells' gene expression profile dynamics using scRNAseq. Bioinformatic analysis identified several pathways that are frequently associated with aging, including senescence, P53, and NF-kB signaling pathways. The authors further explored the potential link of senescence-associated profile with cancer predisposition, specifically focusing on Bcl11b, a regulator of mammary basal stem/progenitor cells shown by the same groups. Bai et al., generated mammary epithelium specific Bcl11b knockout mouse model to examine its role on mammary aging and cancer predisposition, using DMBA treatment. Finally, they used TPCA-1, a NF-kB and Jak-Stat inhibitor, to regulate Bcl11b activity and explore the potential consequence in rejuvenation and cancer predisposition. Taken together, the authors drew the conclusion that mammary basal stem/progenitor cells aging is associated with senescence program, which drive the increased breast cancer risk during aging. Overall, this study presented some interesting observations and informative datasets. However, the reviewer found many overstated conclusions are largely correlative and do not (on many occasions) supported by the data presented.

Specifically:
1/ Authors should be very careful that do not confuse senescence with aging. Senescent cells do accumulate with aging, but it often occurs in many non-aging processes, such as wound healing and tumorigenesis (Oncogene-induced senescence, OIS). A senescent cell is characterized by stable cell cycle arrest, acquisition of SASP, and functional dysregulations. Senescence is not only defined just by gene expression profile OR p16 expression, more importantly, it should be determined on the functional level.
On the conceptual level, the authors often exchange senescence with aging OR mix OIS and aging-associated senescence (DMBM experiment, Figure 3k-n). On the data level, the evidence for the existence of senescence program is weak and sometime contradictory.
For example: a/ what is the functional consequence of senescent mammary basal stem/progenitor cell? The authors must perform in vivo assays to determine, on the single cell level, the functional difference between a senescent mammary basal stem cell and non-senescent counterpart.
b/ Why would mammary cell colony formation capacity increase in the geriatric mice if a senescent cell should seize proliferation both in vitro and in vivo?
c/ Senescence is a cell-intrinsic tumor suppression mechanism as it prevents proliferation in cells with tumorigenic potential. If the Bcl11bKO mammary epithelial cells are senescent and they can't proliferate, how come these cells are more prone to tumorigenic process (Figure 5, line 297-298)?

2/ From the initial bioinformatic analysis, authors classified 4 different cell states based on gene expression profile. Among them, GO analysis indicated cellular senescence is enriched in both state 3 and state 4. However, as the authors rightly pointed out in figure 1, they detected all four states in all age groups, including 2-month-old mice. Actually, the cell

density plot indicates, except geriatric mice, the rest age groups exhibit very similar pattern (Figure 1g, 1h). Why is there such a high percentage of senescent-like cells in the young mammary stem cell population? What would be their functions? In the same time, how do authors reconcile this with increased cancer predisposition in old mice (15-22 month) vs. young? On the technical level, I don't understand Figure 1H. There is a huge difference between 24m and 29m regarding the distribution among 4 states, with State 4 enriched in 24m while no significant difference among states for 29m. Can authors explain this?

3/ It has been reported many times that many pluripotency markers, such as Sox2 and Nanog, are overexpressed in mammary tumors. Can authors provide evidence that, on the single cell level, a senescent mammary stem cell expresses pluripotent stem cell gene, such as Nanog? Co-staining of SAbGal and Nanog on aged mammary section would be sufficient. Otherwise, how could authors be sure that these two processes are actually linked?

4/ There is a lot of speculation and little data to support the mammary phenotypes of Bcl11bKO mice. It is not clear for me what is the role of Bcl11b in mammary stem cell senescence. If Bcl11b is important for inducing senescence in mammary stem cells, then these cells should not proliferate or expand. How do authors explain the expansion of basal compartment in Bcl11b KO (Figure 4)? Detailed functional analysis is required to determine the link between Bcl11b and mammary stem/progenitors' senescence.

Additionally, the SAbGal staining pattern is very different between old mammary gland and Bcl11b KO (Figure 4d). It seems there is a lot of blue cells in the stromal compartment in Bcl11b-KO. What is the cell identity of these blue cells? In addition, there is almost 50% cells are p16+ in 4-months old wild type, while there is no SAbGal staining in the young. How come (Figure 4e)? Authors need to validate the p16 staining with a p16KO mammary gland tissue. It is well known that mouse p16 antibody is not very reliable.

5/ In figure 5, authors compared the tumor latency upon DMBA-induced mammary tumorigenesis assay in WT vs. Bcl11b-KO. The results only support that Bcl11b is potentially a tumor suppressor (Figure 5). There is direct evidence presented here to support the causative link between Bcl11b, senescence, and tumorigenesis. Plus, Figure 3 also showed that DMBA treatment alone also induced senescence program. How come in Figure 5, the WT doesn't resemble "i-Sen" state? Besides, how is the transplantation capacity of Bcl11bKO cells?

6/ Can inhibiting NF-kB pathway rescue Bcl11b KO phenotype? Instead of activation NF-kB pathway in both WT and Bcl11bKO, testing whether diminished NF-kB activity can rescue Bcl11b KO phenotype would provide more stronger and direct evidence to support authors' claims. Besides, what is the expression level of Irak2, Nfkbia, and Nfkb2 in Bcl11bKO mammary cells compared to WT? It would be much more physiological than overexpress Bcl11b.

7/ TPCA-1 is a dual inhibitor of NFkB and Jak/Stat pathway, how can the authors determine the increased cancer latency is not due to reduced inflammation? Authors should include Bcl11bKO mice in the experiment to show the TPCA-1 does not impact tumorigenesis latency in Bcl11bKO. Besides, regarding the experimental design (Figure 7i), shouldn't authors treat mice with TPCA-1 first to reset the cell states first before treating them with DMBA for tumorigenesis?

Reviewer #2 (Remarks to the Author):

The paper describes a thorough investigation of the aging process of a mammary stem cell-enriched population. The study identified cell states and gene programs associated with the aging process. Specifically, the authors focused on a master regulator, Bcl11b, whose under-expression is associated with mammary aging and DMBA-induced tumor formation. The authors showed that the drug TPCA-1 could potentially reverse this process. Overall, the paper provides a thorough investigation of the aging process and tumor formulation of a mammary cell population, with important implications for cancer treatment and prevention.

Overall, the results are significant, the experiments and analyses are appropriate, and the paper is well-written. Specific comments are as follows.

- Relevant literature on single-cell aging. The authors may consider citing and discussing the relevant literature on single-cell aging, including Ximerakis et al. Nature Neuroscience 2019, Kimmel et al. Genome Research 2019, and Zhang et al. eLife 2021.

- A key question of the paper is if the results are specific to the mammary stem cell-enriched population studied in the paper, or if the results can be generalized to other tissues, cell types, and tumor types. For example, can the results of Fig. 3a-d be generalized to other tumor/tissue types in TCGA? Also, interestingly, in Zhang et al. 2021, Bcl11b was reported as an aging gene specific to the "basal cell of epidermis" cell type but was not a global aging gene (Supp. File 3), suggesting the aging programs identified in the paper may be cell type-specific.

- Fig 1c does not support the conclusion that Keratin14 and Keratin 5 expression levels are similar across ages because the tSNE embeddings do not group cells by age. To support this conclusion, scatter plots of gene expression vs. age groups are more appropriate.

- Fig 1d: it is not clear that on the right panel, age aligns with the pseudotime. Showing age as a continuous variable and/or a scatter plot of age vs. pseudotime would be convincing. Also, what's the correlation between age and pseudotime, and can you provide a p-value for significant correlation?

- Fig. 1i: can you provide p-values of the correlation between cell percentage and age in each state? Also, why not have just one figure?

- Line 198: $P<0.05$ will not be significant after multiple testing correction. I suggest applying an FWER/FDR correlation across all p-values computed for each figure.

- Line 261-264: could you provide a p-value for this finding?

- Fig. 7k: can you provide a p-value for the difference?

Reviewer #3 (Remarks to the Author):

This investigation by Bai, et al. focuses on constructing a chronological molecular clock at the level of single-cell transcription, utilizing a cell population enriched with mammary stem

cells, to unravel the dynamics of physiological aging in mice. Their work reveals an asynchronous and progressive process of mammary aging, commencing with an early senescence program characterized by heightened NF-kB and P53 signaling, followed by a disorderly late senescence program marked by diminished NF-kB and P53 signaling. This later phase displays increased PI3K-Akt-mTOR, Wnt, Notch, and pluripotent activity, rendering cells susceptible to cancer predisposition. They found that progression towards the senescence program is governed by the stem cell factor, Bcl11b. In their study, loss of Bcl11b expedited mammary aging and amplified the formation of DMBA-induced tumors. By performing a drug screen, they found a compound, TPCA-1, that can elevate Bcl11b expression, resulting in rejuvenation of mammary cells and curtailed age-related cancer occurrences.

Precise molecular mechanisms that underlie increasing cancer incidence with age have remained elusive, and uncovering those mechanisms is becoming a very active area of investigation. Emerging evidence has suggested that various tissues, and even various cell types within those tissues age at different rates and via differing mechanisms. Limited evidence in the field has also suggested that breast tissue is unique, given the drastic changes it goes through during different stages of life. Given all of this, the current study, which focuses on age-related changes in mammary stem cells, is relatively novel and advances our knowledge in the field. Overall, the study is rigorous and well designed, and experiments are well controlled. There are some necessary clarifications and minor revisions that could potentially strengthen the study, which is already well done.

- The interpretation of the data shown in fig 2, on lines 155-166 could be clarified or even go a step further. It seems the data make an argument for cell abundance, rather than changes in transcriptional programs, underlying the results. Would that be accurate statement? Otherwise, the conclusion that aging is a sophisticated process and not a binary switch (lines 161-163) seems a bit vague.

- Is it also possible that data in Fig 3k-r and described in lines 190-192 might be the consequence of oncogene-induced senescence? Was that explored?

- It was really nice work to follow up the results from the mammary-specific KO of BCL11b (fig 4) with the transplantation experiments (Fig. 5d-f) to control for microenvironmental (or even effects of heterogeneous epithelial cells).

- Given the number of reports that have demonstrated mammary cancer stem cells to be immune-suppressive or immune evasive, I’d be curious to know whether modulation of immune functon(specifically antigen presentation) was observed with modulation of Bcl11b or in the ChIP-seq analysis. Either way, it might serve as an important discussion point to make since there is a notion in the field that cancer stem cells downregulate antigen presentation machinery to promote their progression. Could be interesting to indicate (in Discussion) whether the mammary stem cells in their study do the same or not (or when Bcl11b is modified).

- I agree with the point in lines 402-403 that the data seem to support the notion that carcinogenesis with age is not strictly a matter of accumulating mutations. There are a couple recent reviews (not mine!) that could be cited that elegantly describe that concept: Marongiu and DeGregori, Mol Oncol, 2022 is one example.

- It was helpful to also discuss the study limitations – nicely done.

Response to Reviewers' Comments

We thank all the reviewers for their time and efforts in careful reading and pertinent evaluations. We truly appreciate reviewers' recognition of the significance of our work. Many of the concerns and criticisms brought up by reviewers were genuinely insightful and valuable, which undoubtedly helped us to improve our work. We therefore have revised our work according to reviewers' suggestions and hope the revised version of the manuscript can address reviewers' questions and clarify the confusions.

To make it easier for the reviewers to read, we have labeled the figures in the rebuttal letter as Figure R1-x for reviewer 1, Figure R2-y for reviewer 2, and Figure R3-z for reviewer 3. Point by point responses for reviewers can be found at the following pages:

Reviewer 1: Page 2
Reviewer 2: Page 11
Reviewer 3: Page 15

REVIEWER COMMENTS

Reviewer #1 (Remarks to the Author):

The study by Bai and colleagues aimed to understand the mechanism underlying increased cancer incidence during aging using mammary gland as the primary working model. They characterized the aging-associated mammary basal stem/progenitor cells' gene expression profile dynamics using scRNAseq. Bioinformatic analysis identified several pathways that are frequently associated with aging, including senescence, P53, and NF-kB signaling pathways. The authors further explored the potential link of senescence-associated profile with cancer predisposition, specifically focusing on Bcl11b, a regulator of mammary basal stem/progenitor cells shown by the same groups. Bai et al., generated mammary epithelium specific Bcl11b knockout mouse model to examine its role on mammary aging and cancer predisposition, using DMBA treatment. Finally, they used TPCA-1, a NF-kB and Jak-Stat inhibitor, to regulate Bcl11b activity and explore the potential consequence in rejuvenation and cancer predisposition. Taken together, the authors drew the conclusion that mammary basal stem/progenitor cells aging is associated with senescence program, which drive the increased breast cancer risk during aging. Overall, this study presented some interesting observations and informative datasets. However, the reviewer found many overstated conclusions are largely correlative and do not (on many occasions) supported by the data presented.

We thank the reviewer for these helpful comments that indeed help us to improve our manuscript. For all the concerns raised by the reviewer, we will address them one by one.

Specifically:
1/ Authors should be very careful that do not confuse senescence with aging. Senescent cells do accumulate with aging, but it often occurs in many non-aging processes, such as wound healing and tumorigenesis (Oncogene-induced senescence, OIS). A senescent cell is characterized by stable cell cycle arrest, acquisition of SASP, and functional dysregulations. Senescence is not only defined just by gene expression profile OR p16 expression, more importantly, it should be determined on the functional level.
We thank the reviewer for the helpful comments. We agree with the reviewer that senescence is different from ageing and there are various senescence states associated with various biological processes. We have double checked our manuscript, and corrected those words with misconception. We have highlighted those words in the manuscript (LINE147, 167,168, 281, 299).

In our manuscript, we were mainly focusing on the natural ageing process by collecting cells from various ages of mice. We did not include the wound healing or oncogene induced senescence in our current manuscript. The DMBA induced senescence may cause some confusion. DMBA can indeed induce mutations in oncogenes, however, we speculate that most of the cells should only carry random mutations based on the low tumor frequency. Therefore, DMBA treatment caused senescence is less likely to be OIS, instead, it is rather a DNA damage induced senescence, which also occurs during the natural ageing process. On the single cell transcriptomic level as we have analyzed, DMBA treated cells did not

show a distinct senescence trajectory from the natural senescent cells (Figure R1-1).

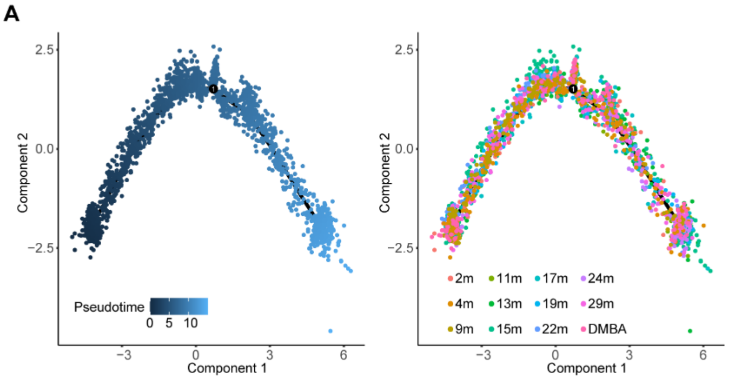

Figure R1-1 Pseudo-time trajectory of mammary cells at various ages and after DMBA treatment

We acknowledged the limitations of relying solely on gene expression to define senescence states. However, utilizing single-cell transcriptomic data to define senescence offers significant advantages. By examining the transcription programs involving thousands of genes, rather than relying on limited markers or phenotypes, we can establish cellular states with greater accuracy. This approach has gradually gained validation in recent years[1-3]. Regarding the limitation of single cell analysis, we have also performed functional assays to validate the senescence phenotype:

1. Secretion Phenotype: We quantified protein expression of IL6 (a typical SASP gene), p-p65(NFκB pathway) and showed consistent results (Fig. 2h-i).
2. Proliferation Phenotype: We quantified the proliferation ability of mammary cells in the organoid culture system and showed that mammary cells from old mice indeed had impaired proliferation. The increased proliferation in the geriatric mice will be explained in the following session (Fig. 3q-r).
3. Regeneration Phenotype: We transplanted young and old mammary cells into syngeneic recipient mice, and showed that old mammary cells indeed were functionally impaired in reconstitution assays. (Extended Data Fig. 5i-j)
4. Molecular perturbation: We deleted the key TF factor Bcl11b and observed increased cells with senescence program, with increased SASP, beta galactosidase activity (Fig. 4d-l), but decreased engraftment ability[4].

Combining our single cell analysis with these functional assays, we think the senescence program we observed in single cell analysis is associated with senescence phenotypes.

However, in this manuscript, we were not able to isolate cells from different states. We have tried hard to isolate the four distinct cell states, but were not able to successfully separate them very well. It is possible we did not find the most appropriate markers to distinguish these cells. But on the other hand, we speculate that the surface markers of cells are limited, and the differences between these cellular states may reside inside the cells. This might be the reason why KO of Bcl11b resulted in the loss of 'q-Young' cells, and TPCA-1 treatment led to reduction of 'd-SEN' while increasing 'a-Young' cells. We hope to be able to separate these cell states in future studies.

On the conceptual level, the authors often exchange senescence with aging OR mix OIS and aging-associated senescence (DMBM experiment, Figure 3k-n). On the data level, the evidence for the existence of senescence program is weak and sometime contradictory.

Thanks for reviewer's helpful comments. We have double checked our manuscript to determine the most appropriated term to use, whether it be 'senescence' or 'ageing'. We have highlighted the words where we revised (LINE147, 167,168, 281, 299).

For example: a/ what is the functional consequence of senescent mammary basal stem/progenitor cell? The authors must perform in vivo assays to determine, on the single cell level, the functional difference between a senescent mammary basal stem cell and non-senescent counterpart. cell should seize proliferation both in vitro and in vivo?

On the single cell level, we found that young mammary stem/progenitor cells were enriched in the pathway of 'DNA replication' 'Oxidative phosphorylation' 'Mismatch repair' indicating higher activity, while old mammary stem/progenitor cells have increased activity in 'NFkb pathway' 'cellular senescence' 'transcriptional misregulation in cancer' as illustrated in Fig. 2a.

We cultured the dissociated mammary stem/progenitor cells from young and old mice. From young to old mice, as the increase of the senescent cells in the stem cell populations, we observed reduced colony formation and proliferation in the 12-month old mammary cells in vitro. The proliferation recovery in 24m mice is likely due to the accumulation of I-SEN cells that have MAPK, PI3K and pluripotent gene activation. In the in vivo transplantation assay, we found that mammary stem/progenitor cells from old mouse had significantly reduced reconstitution ability compared with young mice (Figure R1-2).

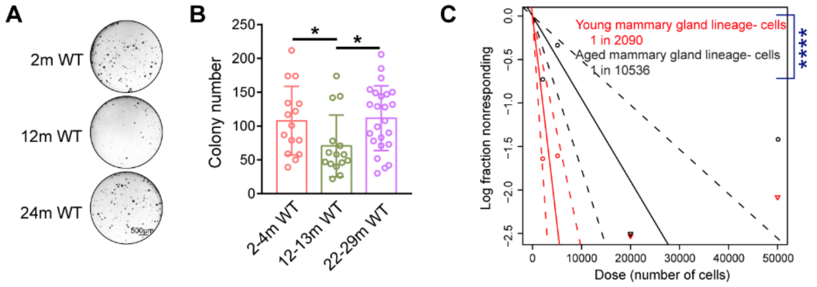

D

Limiting Dilution Transplant of Young and Aged Mammary Gland Lineage- cells

	No. of Injected Cells/Fat Pad	Mammary Tree Outgrowth/No. of Injected Fat Pad	MRU frequency (95% Confidence Interval)
3 month old	50k	4 of 4	1 in 2089 (1 in 1172 to 3723)
	20k	6 of 6	
	5k	8 of 10	
	2k	8 of 10	
18 month old	50k	3 of 4	1 in 10536 (1 in 5675 to 19563)
	20k	6 of 6	
	5k	3 of 10	
	2k	5 of 10	

Figure R1-2 Colony formation and mammary reconstruction from young and old mammary cells.

c/ Senescence is a cell-intrinsic tumor suppression mechanism as it prevents proliferation in cells with tumorigenic potential. If the Bcl11bKO mammary epithelial cells are senescent and they can't

proliferate, how come these cells are more prone to tumorigenic process (Figure 5, line 297-298)?
It is a wonderful question. The senescent cells we identified were heterogeneous. The early senescent cells were proliferation restricted, the typical senescence. However, in the late senescent cells, although the senescence program persisted, there were aberrantly activated oncogenic programs which helped the cells to breach out of the proliferation restriction state. This is supported by our colony formation assay for mammary cells from young, old and geriatric mouse (Figure R1-2). This is also consistent with the finding that in 30-month old mice, there was a reduced p16 expression than 24 month old mice in the single cell analysis (Figure R1-3)[5]. For Bcl11b KO mice, the mammary stem/progenitor cells quickly transit their state to l-SEN state as exemplified in the single cell analysis and were restored with proliferation ability, which facilitate the later oncogenesis.

[Redacted]

Figure R1-3 Cdkn2a expression during ageing (Schaum et al, Nature, 2020)

2/ From the initial bioinformatic analysis, authors classified 4 different cell states based on gene expression profile. Among them, GO analysis indicated cellular senescence is enriched in both state 3 and state 4. However, as the authors rightly pointed out in figure 1, they detected all four states in all age groups, including 2-month-old mice. Actually, the cell density plot indicates, except geriatric mice, the rest age groups exhibit very similar pattern (Figure 1g, 1h). Why is there such a high percentage of senescent-like cells in the young mammary stem cell population? What would be their functions? In the same time, how do authors reconcile this with increased cancer predisposition in old mice (15-22 month) vs. young? On the technical level, I don't understand Figure 1H. There is a huge difference between 24m and 29m regarding the distribution among 4 states, with State 4 enriched in 24m while no significant difference among states for 29m. Can authors explain this?
We are sorry for the confusion. Fig. 1g showed that mice in each age group contained four populations of cells, but the percentage of various populations were very different as exemplified in Fig. 1i. State 1 cells kept decreasing their abundance during aging while state 4 cells kept increasing their abundance. The difference between 24m and 29m is due to the variation between individual mouse. This is what we observed in our data that not all the mice age at the same pace, therefore, even for the same age mice, they showed variations in the composition of the four populations of cells. However, if we group the mice into several age groups, the trend of the decreased young cells and increased senescent cells are clear. We are sorry for not being clear and have revised our text to clarify the point. (LINE 111)

As to why there are some senescent-like cells in the young mammary stem cell population, we think senescent cells are heterogeneous, and not all the senescent cells are 'zombies'.

For example, senescent cells can appear during embryo development process[6,7], and they may also contribute the wound healing and tissue repair process[8-12]. The exact functions of these senescent cells in young mice are indeed an intriguing topic worthy of further investigations.

3/ It has been reported many times that many pluripotency markers, such as Sox2 and Nanog, are overexpressed in mammary tumors. Can authors provide evidence that, on the single cell level, a senescent mammary stem cell expresses pluripotent stem cell gene, such as Nanog? Co-staining of SAbGal and Nanog on aged mammary section would be sufficient. Otherwise, how could authors be sure that these two processes are actually linked?

We thank the reviewer for the wonderful suggestion. SAbGal staining and antibody staining are not compatible with each other and are very challenging to be combined in one staining assay. Therefore, we proposed another experiment and performed staining of OCT4 and p16 staining in the 24-month old consecutive mammary sections. As shown in Figure R1-4, red arrows point to the two corresponding cells in the consecutive sections negative for both p16 and OCT4. Yellow arrows showed mammary cells positive for both p16 and OCT4. We have included this figure in Extended Data Fig. 3f in the revised manuscript.

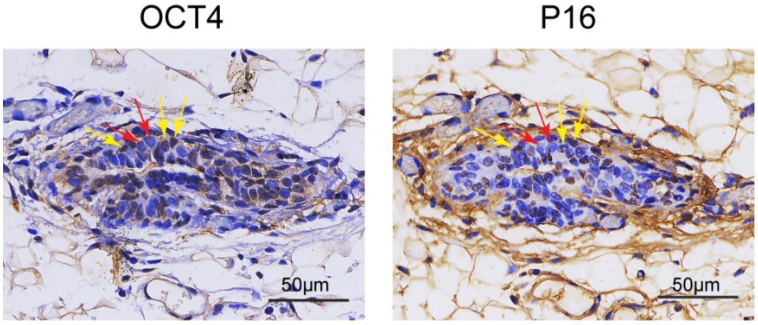

Figure R1-4 Expression of Oct4 and p16 in old mammary gland

4/ There is a lot of speculation and little data to support the mammary phenotypes of Bcl11bKO mice. It is not clear for me what is the role of Bcl11b in mammary stem cell senescence. If Bcl11b is important for inducing senescence in mammary stem cells, then these cells should not proliferate or expand. How do authors explain the expansion of basal compartment in Bcl11b KO (Figure 4)? Detailed functional analysis is required to determine the link between Bcl11b and mammary stem/progenitors' senescence.

We thank the reviewer for the wonderful question. The role of Bcl11b in mammary stem cell senescence was partially reported in our previous paper[4]. Knockout of Bcl11b resulted in exhausted mammary stem cells by balancing p16 and p21 pathways. For the ageing phenotype in stem cells in many other tissues, ageing stem cells frequently showed increased percentage with declined functionality[13-17]. This might be due to the over-proliferation of stem cells at the initial stage and exhaustion at the later stage. We realize that our description of stem cell 'expansion' is not accurate, the percentage increase does not necessarily mean population expansion. We therefore have revised the text to be more precise (LINE 246-254).

Additionally, the SAbGal staining pattern is very different between old mammary gland and Bcl11b

KO (Figure 4d). It seems there is a lot of blue cells in the stromal compartment in Bcl11b-KO. What is the cell identity of these blue cells? In addition, there is almost 50% cells are p16+ in 4-months old wild type, while there is no SAbGal staining in the young. How come (Figure 4e)? Authors need to validate the p16 staining with a p16KO mammary gland tissue. It is well known that mouse p16 antibody is not very reliable.

The senescence phenotype of Bcl11b KO mice was indeed much stronger than the control mice. We reasoned that during the natural ageing process, Bcl11b only slowly decreased its expression and activity, while in the Bcl11b KO mice, Bcl11b expression was abruptly eliminated. This complete elimination caused a much stronger and earlier effect in senescence.

The antibody of p16 that we used has been validated by many other groups and has been cited for about 500 times including some high impact journals listed below:

Braig M, Lee S, Loddenkemper C, Rudolph C, Peters AH, Schlegelberger B, Stein H, Dörken B, Jenuwein T, Schmitt CA. Oncogene-induced senescence as an initial barrier in lymphoma development. ***Nature****. 2005 Aug 4;436(7051):660-5. doi: 10.1038/nature03841. PMID: 16079837.*

Faller WJ, Jackson TJ, Knight JR, Ridgway RA, Jamieson T, Karim SA, Jones C, Radulescu S, Huels DJ, Myant KB, Dudek KM, Casey HA, Scopelliti A, Cordero JB, Vidal M, Pende M, Ryazanov AG, Sonenberg N, Meyuhas O, Hall MN, Bushell M, Willis AE, Sansom OJ. mTORC1-mediated translational elongation limits intestinal tumour initiation and growth. ***Nature.*** *2015 Jan 22;517(7535):497-500. doi: 10.1038/nature13896. Epub 2014 Nov 5. PMID: 25383520*

Huang L, Desai R, Conrad DN, Leite NC, Akshinthala D, Lim CM, Gonzalez R, Muthuswamy LB, Gartner Z, Muthuswamy SK. Commitment and oncogene-induced plasticity of human stem cell-derived pancreatic acinar and ductal organoids. ***Cell Stem Cell.*** *2021 Jun 3;28(6):1090-1104.e6. doi: 10.1016/j.stem.2021.03.022. Epub 2021 Apr 28. PMID: 33915081*

We therefore think the antibody is a reliable one for detection of p16. The discrepancy between p16 and beta-gal staining could be that p16 expression is the early stage of senescence before beta gal staining phenotype. At the same time, we realize that beta gal staining intensity is also developing-time dependent, there might be some weak beta gal positive cells in young tissues that have been omitted due to the insufficient time of developing.

5/ In figure 5, authors compared the tumor latency upon DMBA-induced mammary tumorigenesis assay in WT vs. Bcl11b-KO. The results only support that Bcl11b is potentially a tumor suppressor (Figure 5). There is direct evidence presented here to support the causative link between Bcl11b, senescence, and tumorigenesis. Plus, Figure 3 also showed that DMBA treatment alone also induced senescence program. How come in Figure 5, the WT doesn't resemble "i-Sen" state? Besides, how is the transplantation capacity of Bcl11bKO cells?

We think it is possible that Bcl11b's roles are pleiotropic including acting as a tumor suppressor. In our original manuscript, we showed that old mammary cells gave rise to

more tumors than young mammary cells after DMBA treatment. In addition, KO of Bcl11b resulted in activation of senescence program, and increased tumor formation after DMBA treatment. We also showed that the senescence caused by Bcl11b KO can be inhibited by the downstream pathway inhibition (TPCA-1)(Extended Data Fig 12). We have also shown that DMBA triggered senescence in the mammary gland can be reversed by TPCA-1 treatment, we found tumor incidence was clearly reduced accordingly (Fig. 3k-p and 7i-k). This suggests that modulation of the senescence program can influence the ageing associated tumor formation. These data collectively support the hypothesis that Bcl11b KO resulted in mammary cell senescence which was able to promote tumor initiation.

Bcl11b KO cell transplantation has been reported in our previous paper[4]. Knockout of Bcl11b caused stem cell exhaustion and reduced engraftment rate in the transplantation assay (Figure R1-5).

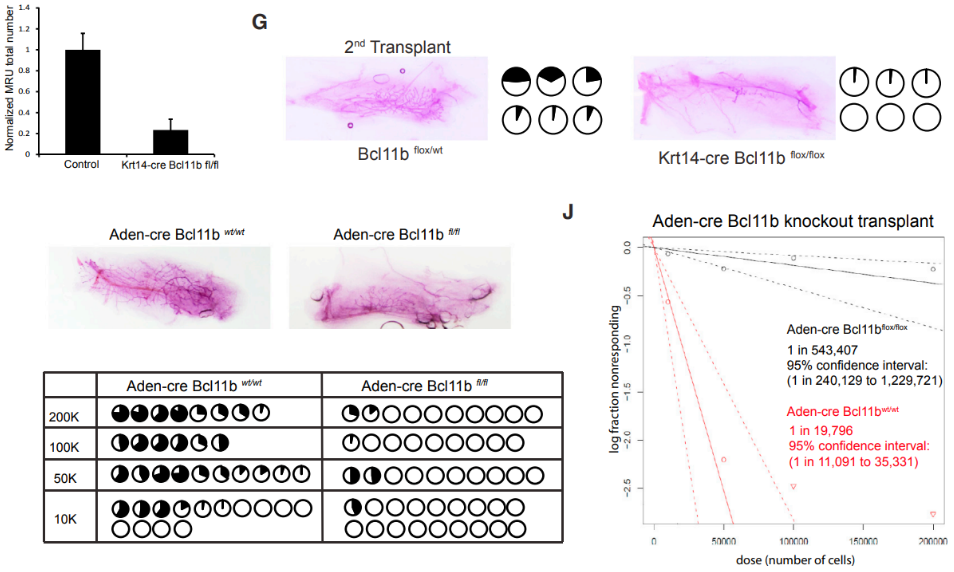

Figure R1-5 Knockout of Bcl11b resulted in mammary stem cell exhaustion

6/ Can inhibiting NF-kB pathway rescue Bcl11b KO phenotype? Instead of activation NF-kB pathway in both WT and Bcl11bKO, testing whether diminished NF-kB activity can rescue Bcl11b KO phenotype would provide more stronger and direct evidence to support authors' claims. Besides, what is the expression level of Irak2, Nfkbia, and Nfkb2 in Bcl11bKO mammary cells compared to WT? It would be much more physiological than overexpress Bcl11b.

Thanks for the great question. We performed the NFkb inhibition assay in Bcl11b KO mice and analyzed by single cell RNAseq as shown in Extended Data Fig. 13 of the revised manuscript (Figure R1-6). As we can see, Nfkb pathway activity was clearly inhibited by BMS-345541. However, the 'JAK-STAT pathway', 'cellular senescence', 'SASP gene', remained comparable to Bcl11b KO cells. In addition, 'Bcl11b activity' was not rescued by the BMS-345541 treatment. The 'MAPK signaling' and 'PI3K pathway', which are frequently linked in cancer initiation were not inhibited by BMS-345541. These analyses collectively suggest that inhibition of Nfkb pathway alone is not sufficient to rescue Bcl11b KO phenotype. We have incorporated the data in Extended Data Fig. 13 and Text Line 374 of the revised manuscript.

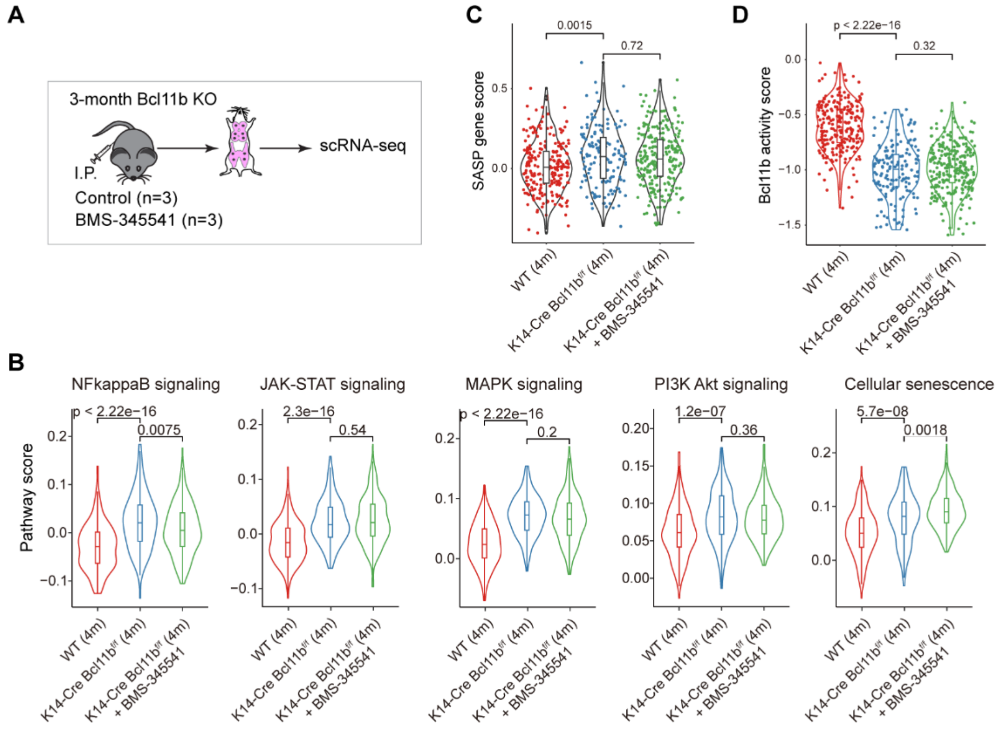

Figure R1-6 inhibition of Nfkb alone does not rescue Bcl11b KO phenotypes

As to the Nfkb related gene expression, we are showing the expression of Irak2, Nfkbia, Nfkb2, Nfkb1 expression below. The canonical Nfkb pathway genes were all upregulated upon Bcl11b knockout.

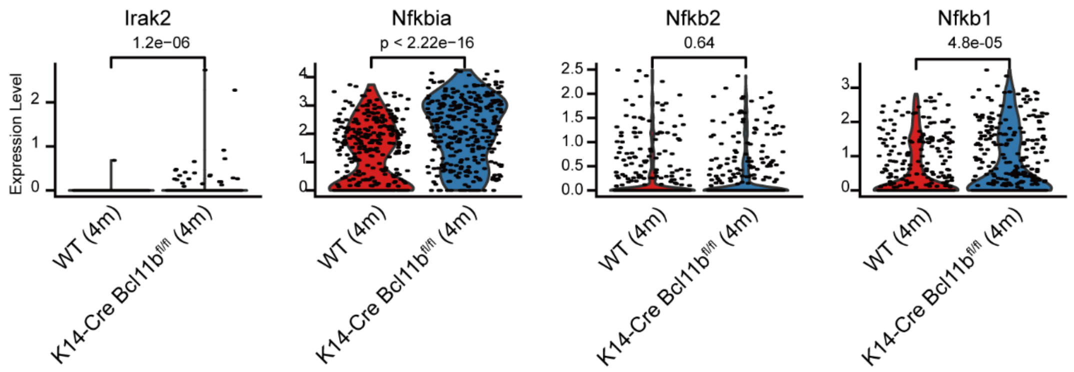

Figure R1-7 Expression of Nfkb pathway factors after Bcl11b KO

7/ TPCA-1 is a dual inhibitor of NFkB and Jak/Stat pathway, how can the authors determine the increased cancer latency is not due to reduced inflammation? Authors should include Bcl11bKO mice in the experiment to show the TPCA-1 does not impact tumorigenesis latency in Bcl11bKO. Besides, regarding the experimental design (Figure 7i), shouldn't authors treat mice with TPCA-1 first to reset the cell states first before treating them with DMBA for tumorigenesis?

We do not claim that the increased latency is not due to reduced inflammation. Since senescence cells will secrete SASPs which can trigger inflammation. TPCA-1 treatment will reduce SASP and therefore reduce inflammation. TPCA-1 treatment may have systemic effect, which is a common problem for all the drug treatment assays. This is why

we did the experiment of tumor induction in transplanted WT and Krt14-cre Bcl11b$^{f/f}$ cells to show the tumor cell intrinsic effect (Fig. 5d-h).

We are not able to use Bcl11b KO mice to perform the tumor induction assay because Bcl11b KO mice were sick and cannot survive longer than 16 weeks.

We have thought about the TPCA-1 treatment strategies. We reasoned that TPCA-1 treatment followed by DMBA treatment would cause different mutation burdens as TPCA-1 can change cell fate. Then it will be the mutation load that determines the final outcome instead of senescence state. DMBA treatment first will ensure similar mutation burden at the beginning, and the final cancer latency result will be possibly interpreted as the TPCA-1 mediated cell fate changes.

Reviewer #2 (Remarks to the Author):

The paper describes a thorough investigation of the aging process of a mammary stem cell-enriched population. The study identified cell states and gene programs associated with the aging process. Specifically, the authors focused on a master re gulator, Bcl11b, whose under-expression is associated with mammary aging and DMBA-induced tumor formation. The authors showed that the drug TPCA-1 could potentially reverse this process. Overall, the paper provides a thorough investigation of the aging process and tumor formulation of a mammary cell population, with important implications for cancer treatment and prevention. Overall, the results are significant, the experiments and analyses are appropriate, and the paper is well-written. Specific comments are as follows.

We truly thank the reviewer for these helpful comments. We will address the concerns one by one below:

- Relevant literature on single-cell aging. The authors may consider citing and discussing the relevant literature on single-cell aging, including Ximerakis et al. Nature Neuroscience 2019, Kimmel et al. Genome Research 2019, and Zhang et al. eLife 2021.

We thank the reviewer for kind suggestion. We have discussed and cited these references in the revised manuscript in TEXT line 429.

- A key question of the paper is if the results are specific to the mammary stem cell-enriched population studied in the paper, or if the results can be generalized to other tissues, cell types, and tumor types. For example, can the results of Fig. 3a-d be generalized to other tumor/tissue types in TCGA? Also, interestingly, in Zhang et al. 2021, Bcl11b was reported as an aging gene specific to the "basal cell of epidermis" cell type but was not a global aging gene (Supp. File 3), suggesting the aging programs identified in the paper may be cell type-specific.

We thank the reviewer for the wonderful question. We have performed the suggested analysis in TCGA database, we found across various tissues/tumors, Bcl11b's activity (indicated by its ChIP-seq targets) is frequently reduced in tumors compared with normal tissues (Figure R2-1). Consistent with this data, the related senescence pathways, oncogenic factors and pathways are also elevated in tumors compared with normal tissues. We recognize that in Zhang et al's paper, Bcl11b was a specific ageing gene in basal cell of epidermis. Although Bcl11b may not be a global ageing gene, we think Bcl11b's down stream pathways might be conserved in the ageing process across various tissues. However, it requires comprehensive studies to robustly demonstrate this point. We have discussed this point in TEXT line 429 to clarify the limitations of our study.

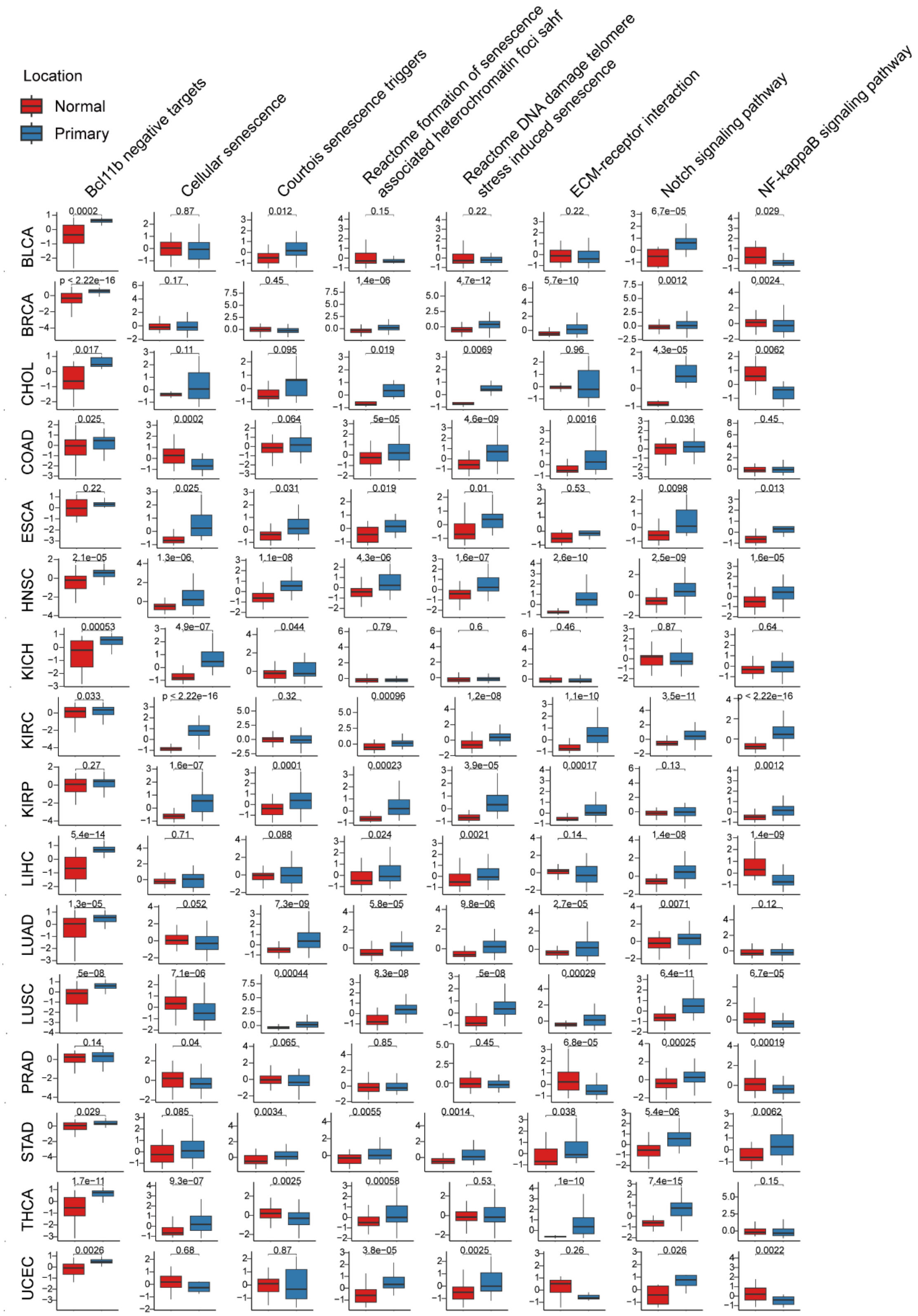

Figure R2-1 Pathway and activity analysis across various cancer types

- Fig 1c does not support the conclusion that Keratin14 and Keratin 5 expression levels are similar across ages because the tSNE embeddings do not group cells by age. To support this conclusion, scatter plots of gene expression vs. age groups are more appropriate.

We thank the reviewer for the kind suggestion. We have re-analyzed the expression of Krt14 and Krt5 along the age (Figure R2-2). The expression of these two genes remained at similar level from young to old mice, but slightly decreased in the geriatric mice. We have revised our statement in the revised manuscript accordingly in TEXT line 93, and incorporated the figure into Extended Data Fig.1g.

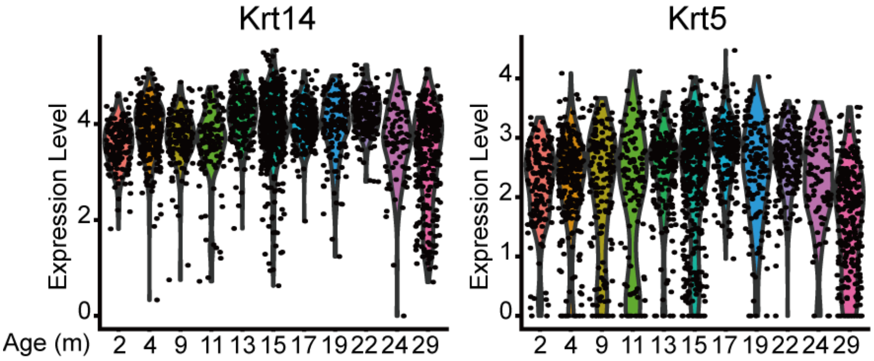

Figure R2-2 Krt14 and Krt5 expression in mammary cells across various mouse ages

- Fig 1d: it is not clear that on the right panel, age aligns with the pseudotime. Showing age as a continuous variable and/or a scatter plot of age vs. pseudotime would be convincing. Also, what's the correlation between age and pseudotime*, and can you provide a p-value for significant correlation?*

We thank the reviewer for this nice suggestion. Now we are providing the association between age and pseudotime. We performed linear regression between the average pseudotime of each mouse age and the real mouse age, and found that they are correlated with each other (R=0.61, p<0.05)(Figure R2-3) (Extended Data Fig. 1f). This suggests that the pseudotime value can be used as an indication of the mouse age.

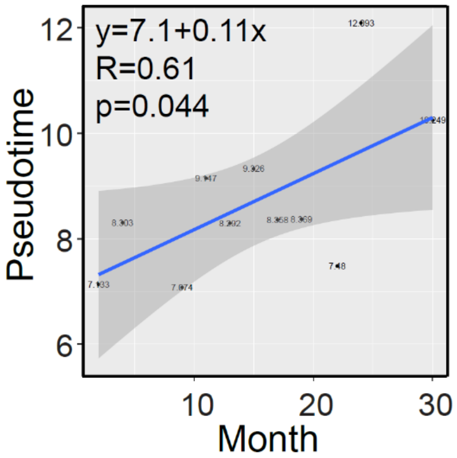

Figure R2-3 Correlation between pseudotime and mouse age

- Fig. 1i: can you provide p-values of the correlation between cell percentage and age in each state? Also, why not have just one figure?

We have included the p value (State 1: p=0.0052; State 2: p=0.73; State 3: p=0.72; State 4: p=0.05;) in the Extended Data Fig. 1e and combined the trend lines into one panel (Fig. 1i).

- Line 198: P<0.05 will not be significant after multiple testing correction. I suggest applying an FWER/FDR correlation across all p-values computed for each figure.

Thanks for the great suggestion. We have performed statistical analysis as suggested by the reviewer. And they all showed statistical significance (Figure R2-4).

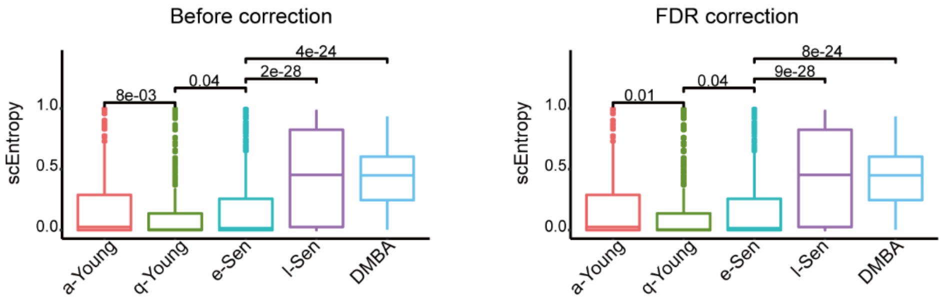

Figure R2-4 Single Cell signaling entropy in various cell states

- Line 261-264: could you provide a p-value for this finding?

We have performed Chi-squared test and the p value is 0.0000002947, we have revised the text in Line 264.

- Fig. 7k: can you provide a p-value for the difference?

We have performed paired t test and the p value p<0.05.

Reviewer #3 (Remarks to the Author):

This investigation by Bai, et al. focuses on constructing a chronological molecular clock at the level of single-cell transcription, utilizing a cell population enriched with mammary stem cells, to unravel the dynamics of physiological aging in mice. Their work reveals an asynchronous and progressive process of mammary aging, commencing with an early senescence program characterized by heightened NF-kB and P53 signaling, followed by a disorderly late senescence program marked by diminished NF-kB and P53 signaling. This later phase displays increased PI3K-Akt-mTOR, Wnt, Notch, and pluripotent activity, rendering cells susceptible to cancer predisposition. They found that progression towards the senescence program is governed by the stem cell factor, Bcl11b. In their study, loss of Bcl11b expedited mammary aging and amplified the formation of DMBA-induced tumors. By performing a drug screen, they found a compound, TPCA-1, that can elevate Bcl11b expression, resulting in rejuvenation of mammary cells and curtailed age-related cancer occurrences.

Precise molecular mechanisms that underlie increasing cancer incidence with age have remained elusive, and uncovering those mechanisms is becoming a very active area of investigation. Emerging evidence has suggested that various tissues, and even various cell types within those tissues age at different rates and via differing mechanisms. Limited evidence in the field has also suggested that breast tissue is unique, given the drastic changes it goes through during different stages of life. Given all of this, the current study, which focuses on age-related changes in mammary stem cells, is relatively novel and advances our knowledge in the field. Overall, the study is rigorous and well designed, and experiments are well controlled. There are some necessary clarifications and minor revisions that could potentially strengthen the study, which is already well done.

We thank the reviewer for these helpful comments that indeed help us to improve our manuscript. For all the concerns raised by the reviewer, we will address them one by one.

- The interpretation of the data shown in fig 2, on lines 155-166 could be clarified or even go a step further. It seems the data make an argument for cell abundance, rather than changes in transcriptional programs, underlying the results. Would that be accurate statement? Otherwise, the conclusion that aging is a sophisticated process and not a binary switch (lines 161-163) seems a bit vague.

We thank the reviewer for the kind suggestion. We think the difference between young and old mammary cells is the relative abundance of various types of cells, and the ageing process is accompanied with altered homeostasis among four different cellular states. The various signaling pathways just determine the identity and the transition of different cell states. We have revised our statement to precisely describe the phenotype in the TEXT line 164 of the revised manuscript.

- Is it also possible that data in Fig 3k-r and described in lines 190-192 might be the consequence of oncogene-induced senescence? Was that explored?

We found that DMBA triggered senescence mainly increased the e-SEN state cells as quantified in Fig. 3m. We also analyzed the oncogene expression level after DMBA treatment, and found that most of the oncogenes do not show significant difference. WE

speculate that DMBA mainly causes DNA damages and promotes random mutations, and some of the targeted cells happen to harbor the oncogene mutations. Therefore, for the majority of the treated cells, we speculate the senescence program triggered by DMBA is possibly caused by the activation of DNA damage repair activity instead of oncogene induced senescence.

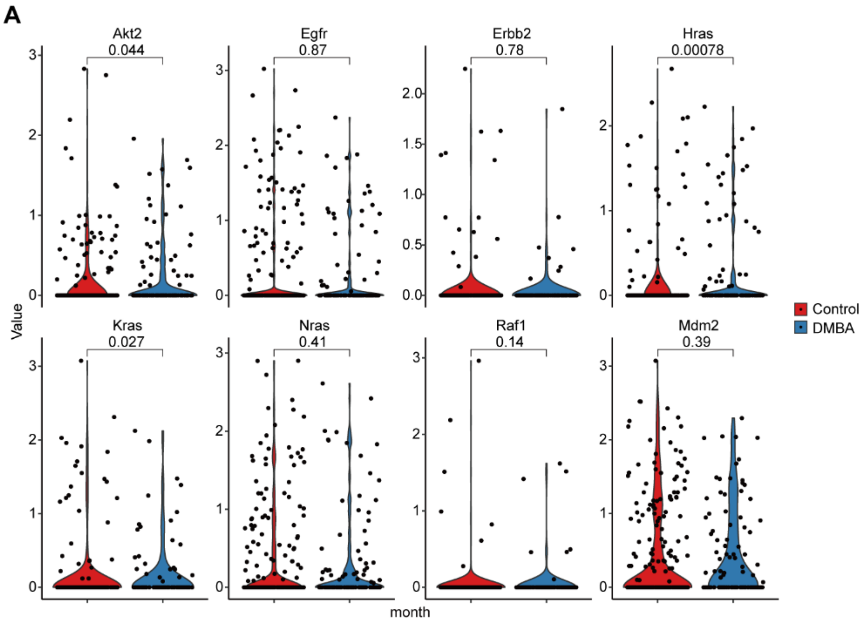

Figure R3-1 Oncogene expression analysis after DMBA treatment

- It was really nice work to follow up the results from the mammary-specific KO of BCL11b (fig 4) with the transplantation experiments (Fig. 5d-f) to control for microenvironmental (or even effects of heterogeneous epithelial cells).

Thanks!

- Given the number of reports that have demonstrated mammary cancer stem cells to be immune-suppressive or immune evasive, I'd be curious to know whether modulation of immune functon(specifically antigen presentation) was observed with modulation of Bcl11b or in the ChIP-seq analysis. Either way, it might serve as an important discussion point to make since there is a notion in the field that cancer stem cells ***downregulate antigen presentation machinery to promote their progression****. Could be interesting to indicate (in Discussion) whether the mammary stem cells in their study do the same or not (or when Bcl11b is modified).*

Thanks for the great suggestion. We have compared the wt and Bcl11b KO cells for the immune related pathways and found that the PD-L1 and PD-1 checkpoint pathway was elevated after Bcl11b deletion, which is consistent with reviewer's hypothesis (Figure R3-2). However, in the CHIPseq data, we did not identify any targets involved in this PD-L1 and PD1 pathway. Therefore, the regulation between Bcl11b and PD-L1 and PD1 pathway might be indirect. We have included this data in the Extended Data Fig. 8e and discussed about it in TEXT line 300 of the revised manuscript.

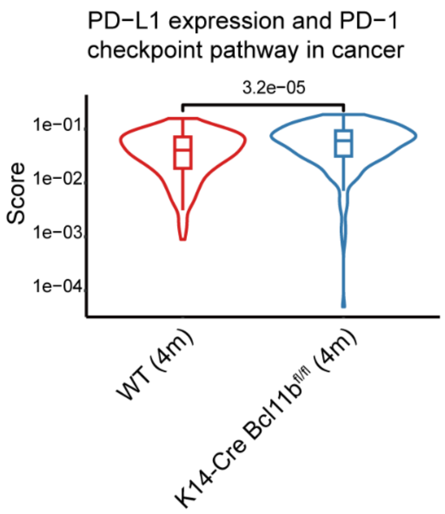

Figure R3-2 PDL1 PD1 pathway analysis in wt and Bcl11b mutant cells

- I agree with the point in lines 402-403 that the data seem to support the notion that carcinogenesis with age is not strictly a matter of accumulating mutations. There are a couple recent reviews (not mine!) that could be cited that elegantly describe that concept: Marongiu and DeGregori, Mol Oncol, 2022 is one example.

Thanks for the suggestion and we have included the reference in the revised manuscript in TEXT line 415.

- It was helpful to also discuss the study limitations – nicely done.

Thanks!

References

1 A single-cell transcriptomic atlas characterizes ageing tissues in the mouse. *Nature* **583**, 590-595, doi:10.1038/s41586-020-2496-1 (2020).

2 Saul, D. *et al.* A new gene set identifies senescent cells and predicts senescence-associated pathways across tissues. *Nat Commun* **13**, 4827, doi:10.1038/s41467-022-32552-1 (2022).

3 O'Sullivan, E. D. *et al.* Single-cell analysis of senescent epithelia reveals targetable mechanisms promoting fibrosis. *JCI insight* **7**, doi:10.1172/jci.insight.154124 (2022).

4 Cai, S. *et al.* A Quiescent Bcl11b High Stem Cell Population Is Required for Maintenance of the Mammary Gland. *Cell Stem Cell* **20**, 247-260.e245, doi:10.1016/j.stem.2016.11.007 (2017).

5 Schaum, N. *et al.* Ageing hallmarks exhibit organ-specific temporal signatures. *Nature* **583**, 596-602, doi:10.1038/s41586-020-2499-y (2020).

6 Storer, M. *et al.* Senescence is a developmental mechanism that contributes to embryonic growth and patterning. *Cell* **155**, 1119-1130, doi:10.1016/j.cell.2013.10.041 (2013).

7 Muñoz-Espín, D. *et al.* Programmed cell senescence during mammalian embryonic development. *Cell* **155**, 1104-1118, doi:10.1016/j.cell.2013.10.019 (2013).

8 Jun, J. I. & Lau, L. F. The matricellular protein CCN1 induces fibroblast senescence and restricts fibrosis in cutaneous wound healing. *Nature cell biology* **12**, 676-685, doi:10.1038/ncb2070 (2010).

9 Demaria, M. *et al.* An essential role for senescent cells in optimal wound healing through secretion of PDGF-AA. *Developmental cell* **31**, 722-733, doi:10.1016/j.devcel.2014.11.012 (2014).

10 Ritschka, B. *et al.* The senescence-associated secretory phenotype induces cellular plasticity and tissue regeneration. *Genes & development* **31**, 172-183, doi:10.1101/gad.290635.116 (2017).

11 Mosteiro, L. *et al.* Tissue damage and senescence provide critical signals for cellular reprogramming in vivo. *Science (New York, N.Y.)* **354**, doi:10.1126/science.aaf4445 (2016).

12 Chiche, A. *et al.* Injury-Induced Senescence Enables In Vivo Reprogramming in Skeletal Muscle. *Cell Stem Cell* **20**, 407-414.e404, doi:10.1016/j.stem.2016.11.020 (2017).

13 Schultz, M. B. & Sinclair, D. A. When stem cells grow old: phenotypes and mechanisms of stem cell aging. *Development (Cambridge, England)* **143**, 3-14, doi:10.1242/dev.130633 (2016).

14 Pang, W. W., Schrier, S. L. & Weissman, I. L. Age-associated changes in human hematopoietic stem cells. *Seminars in hematology* **54**, 39-42, doi:10.1053/j.seminhematol.2016.10.004 (2017).

15 Jamieson, C. H. M. & Weissman, I. L. Stem-Cell Aging and Pathways to Precancer Evolution. *The New England journal of medicine* **389**, 1310-1319, doi:10.1056/NEJMra2304431 (2023).

16 Rossi, D. J., Jamieson, C. H. & Weissman, I. L. Stems cells and the pathways to aging and cancer. *Cell* **132**, 681-696, doi:10.1016/j.cell.2008.01.036 (2008).

17 Dong, Q. *et al.* Aging is associated with an expansion of CD49f(hi) mammary stem cells that show a decline in function and increased transformation potential. *Aging* **8**, 2754-2776, doi:10.18632/aging.101082 (2016).

REVIEWER COMMENTS

Reviewer #1 (Remarks to the Author):

I appreciate the efforts made to clarify and improve the manuscripts. Although certain points are being addressed, there are several key points remain to be experimentally addressed.

1/ The functional consequence of senescent mammary basal stem/progenitor cells.

The authors compared the young mammary stem/progenitor cells to the old ones using colony formation assay. However, this comparison will only reflect, on the population level, mammary stem/progenitor cells functionality decline during aging, which is something well documented previously. The right comparison should be separating senescent and non-senescent mammary stem/progenitor cells from the same age and compare their functionality, using assays such as Spider-(betal)-Gal (Moiseeva et al. Nature 2023) or p16 reporter mouse models (INKBRITE, NS Reyes et al. Science 2023). This comparison is particularly important in young and very old mice where the authors observed interesting p16 expression pattern.

2/ Why would senescent Bcl11bKO mammary epithelial cells be more prone to tumorigenic process (Figure 5). Page 5

Do authors have any experimental data support the comments made answering this question (page 5), as “in the late senescent cells, although the senescence program persisted, there were aberrantly activated oncogenic programs which helped the cells to breach out of the proliferation restriction state.”? This would imply that senescent cells can be reverted to proliferation, which is highly controversial in the senescence field. The other possibility is that what authors observed are due to non-cell-autonomous effect of senescence, where senescent Bcl11bKO cells promote non-senescent cells tumorigenesis. The authors should experimentally address this possibility.

3/ p16 antibody.

It is absolutely essential for authors to validate their p16 staining using p16-KO mammary gland. It is well known in the field that p16 antibody is highly tissue-specific, even the same p16 antibody could detect non-specific staining in different tissues. It is important to ensure the reproducibility of the findings. Besides, the authors used p16 and OCT4 co-staining to address my point about pluripotency markers and senescent cells (page 6), it is a circular argument at this point.

If the authors can’t find/use the p16-KO mice, the minimal required validation is quantitative RNA-ISH, such as RNAscope.

Reviewer #2 (Remarks to the Author):

I thank the authors for revising the paper and for the detailed response. My follow-up comments are as follows.

- A key question of the paper is if the results are specific to the mammary stem cell-enriched population studied in the paper, or if the results can be generalized to other tissues, cell types, and tumor types. For example, can the results of Fig. 3a-d be generalized to other tumor/tissue types in TCGA? Also, interestingly, in Zhang et al. 2021, Bcl11b was reported as an aging gene specific to the "basal cell of epidermis" cell type but was not a global aging gene (Supp. File 3), suggesting the aging programs identified in the paper may be cell type-specific.

Rv1: The response makes sense, but the current discussion in line 429 is very short. I suggest that the authors incorporate the response into the discussion in the main paper.

- Fig. 1i: can you provide p-values of the correlation between cell percentage and age in each state? Also, why not have just one figure?
Rv1: please include the p-values in the main text because the readers would want to know the statistical significance of the evidence supporting your claim. Also, p=0.05 is not significant after multiple testing correction (e.g., p=0.05 > 0.05/4). Therefore, I would be cautious about the claim that "that of the State 4 cells continuously increased with age."

- Line 261-264: could you provide a p-value for this finding?
Rv1: p=0.0000002947>0.0000001, contradictory to the updated text.

- Fig. 7k: can you provide a p-value for the difference?
Rv1: where did you report this p-value in the updated text?

Reviewer #3 (Remarks to the Author):

The authors have provided additional supporting and control data, provided clarifying statements, toned-down overstatements, and added important discussion points that together, significantly strengthen the manuscript. Their study remains relatively novel and advances the rapidly growing field of aging and cancer. Well done.

Response to Reviewers' Comments

Reviewer #1:

1/ The functional consequence of senescent mammary basal stem/progenitor cells.

The authors compared the young mammary stem/progenitor cells to the old ones using colony formation assay. However, this comparison will only reflect, on the population level, mammary stem/progenitor cells functionality decline during aging, which is something well documented previously. The right comparison should be separating senescent and non-senescent mammary stem/progenitor cells from the same age and compare their functionality, using assays such as Spider-(betal)-Gal (Moiseeva et al. Nature 2023) or p16 reporter mouse models (INKBRITE, NS Reyes et al. Science 2023). This comparison is particularly important in young and very old mice where the authors observed interesting p16 expression pattern.

Response: We thank the reviewer for the kind suggestion. The best experiment to demonstrate the consequence of senescent mammary stem cells is to separate the four different states and test their functionalities individually. Unfortunately, we were not able to find the appropriate marker to do it. As suggested by the reviewer, Beta-Gal assay and p16 expression are good senescence markers. Although it is not clear how much these individual markers represent the heterogeneity of the senescent populations, we performed the suggested experiment using SPiDER detection kit. We stained the mammary cells from 2month, 11month and 22month mice following the manufacturer's instructions, and found that most of mammary stem cells were SPiDER+ compared with the negative control. The distribution of SPiDER staining was similar among different ages. We were not able to identify the true SPiDER- cells. We therefore sorted the top 10% of the SPiDER high cells from mammary cells of various ages and tested their colony formation ability. We found that these SPiDER high cells showed an increased ability in colony formation in the 22month mice (**Figure R1-1**). This is consistent with our hypothesis that in the geriatric mice, the senescent cells have partially recovered their proliferation ability due to the reactivation of many cancer-related pathways.

However, we do have concerns about the conclusion drawn from any individual senescence markers, as we believe the senescent cells during ageing are heterogeneous. The beta-gal or p16 may label certain senescent populations but may not represent the whole senescent cells. That is why we did single cell transcriptomic analysis to build the molecular dynamics during ageing. We hope in the future we can develop better senescent cell reporter that can truly identify the various senescent states we discovered.

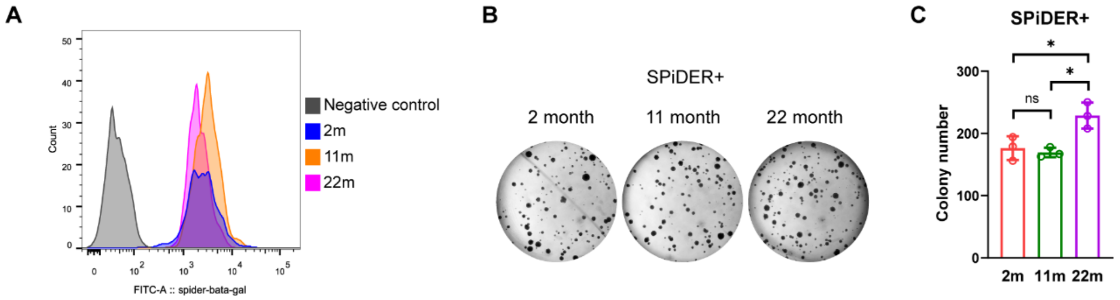

Figure R1-1 Colony formation ability of SPiDER positive CD49f^{high}EpCAM^{low} mammary gland cells. (A) Representative FACS plot of SPiDER positive cells in CD49f^{high}EpCAM^{low} mammary gland cells from 2m, 11m and 22m mice. (B-C) Representative images (B) and quantification (C) of colony formation assay of SPiDER+ CD49f^{high}EpCAM^{low} mammary cells from 2m, 11m and 22m old WT mammary gland. Statistical analysis was performed using two-tailed unpaired t-tests; data are presented as mean ± SD; *P<0.05.

2/ Why would senescent Bcl11bKO mammary epithelial cells be more prone to tumorigenic process (Figure 5). Page 5 Do authors have any experimental data support the comments made answering this question (page 5), as "in the late senescent cells, although the senescence program persisted, there were aberrantly activated oncogenic programs which helped the cells to breach out of the proliferation restriction state."? This would imply that senescent cells can be reverted to proliferation, which is highly controversial in the senescence field. The other possibility is that what authors observed are due to non-cell-autonomous effect of senescence, where senescent Bcl11bKO cells promote non-senescent cells tumorigenesis. The authors should experimentally address this possibility.

Response: We appreciate reviewer's alternative explanation. In our previous Cell Stem Cell paper, we tested the proliferation ability of Bcl11b KO cells, the majority of which are late senescent cells[1]. We indeed found Bcl11b KO cells proliferate faster than wt cells (**Figure R1-2**). As to the possibility of non-cell autonomous effect of senescence, our data do not support this hypothesis, because we performed cancer initiation assay by transplanting combined WT young cells and WT old cells, the tumor outgrowths were always more from old cells instead of young cells (Figure 5A-C from the revised manuscript). In addition, when we transplanted wt cells and Bcl11b KO cells into same host mice, the tumors again were initiated more from Bcl11b KO cells (Figure 5D-F from the revised manuscript). In the future, we hope to be able to trace those late senescent cells to definitively demonstrate this point. We have discussed this limitation in the discussion session in TEXT line 436-445 of the revised manuscript.

[Redacted]

Figure R1-2 Bcl11b KO cells proliferate faster than wt cells[1].

3/ p16 antibody.

It is absolutely essential for authors to validate their p16 staining using p16-KO mammary gland. It is well known in the field that p16 antibody is highly tissue-specific, even the same p16 antibody could detect non-specific staining in different tissues. It is important to ensure the reproducibility of the findings. Besides, the authors used p16 and OCT4 co-staining to address my point about pluripotency markers and senescent cells (page 6), it is a circular argument at this point.

If the authors can't find/use the p16-KO mice, the minimal required validation is quantitative RNA-ISH, such as RNAscope.

Response: We thank reviewer's suggestion. We have performed p16 KO in the MMTV-PyMT breast cancer cells, and stained the cells with the p16 antibody used in our manuscript. We indeed observed significantly reduced fluorescence after p16 ko (**Figure R1-3**), which validate the specificity of this antibody.

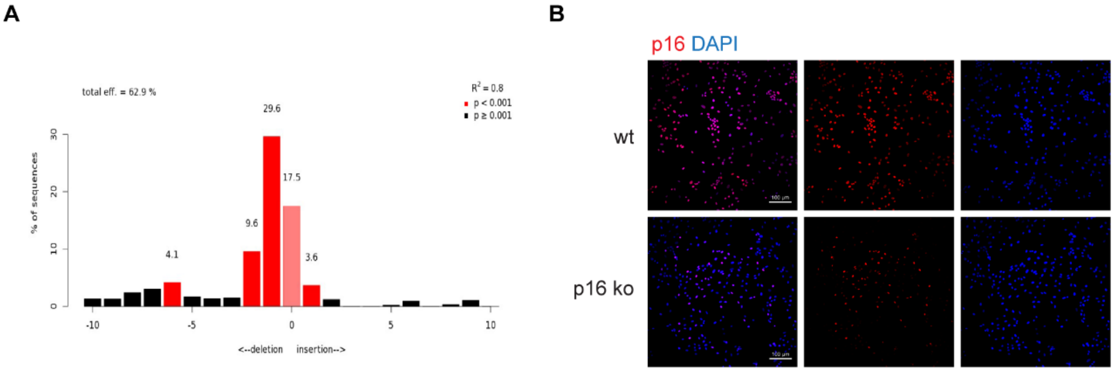

Figure R1-3 p16^{Ink4a} staining in wt and CRISPR/Cas9 based p16 ko cells. (A) Indel frequencies within ±10 bp from theoretical gRNA breakpoints. Total editing efficiency = 62.9%. (B) Representative p16^{Ink4a} staining images in wt and p16 ko MMTV-PyMT breast cancer cells. Scale bar, 100 μm.

Reviewer #2:

I thank the authors for revising the paper and for the detailed response. My follow-up comments are as follows.

- A key question of the paper is if the results are specific to the mammary stem cell-enriched population studied in the paper, or if the results can be generalized to other tissues, cell types, and tumor types. For example, can the results of Fig. 3a-d be generalized to other tumor/tissue types in TCGA? Also, interestingly, in Zhang et al. 2021, Bcl11b was reported as an aging gene specific to the "basal cell of epidermis" cell type but was not a global aging gene (Supp. File 3), suggesting the aging programs identified in the paper may be cell type-specific.
Rv1: The response makes sense, but the current discussion in line 429 is very short. I suggest that the authors incorporate the response into the discussion in the main paper.

Response:
We thank the reviewer for the suggestion. We have elaborated the discussion in TEXT line 427-434 in the revised manuscript.

- Fig. 1i: can you provide p-values of the correlation between cell percentage and age in each state? Also, why not have just one figure?
Rv1: please include the p-values in the main text because the readers would want to know the statistical significance of the evidence supporting your claim. Also, p=0.05 is not significant after multiple testing correction (e.g., p=0.05 > 0.05/4). Therefore, I would be cautious about the claim that "that of the State 4 cells continuously increased with age."
Response: Thanks for your suggestion! We have revised the text accordingly in Line 110.

- Line 261-264: could you provide a p-value for this finding?
Rv1: p=0.0000002947>0.0000001, contradictory to the updated text.
Response: Thanks! We have revised the text in Line 264, p<0.000001.

- Fig. 7k: can you provide a p-value for the difference?
Rv1: where did you report this p-value in the updated text?
Response: We have revised the text in Line 388 and figure legend of Fig.7k.

REFERENCE

1 Cai, S. *et al.* A Quiescent Bcl11b High Stem Cell Population Is Required for Maintenance of the Mammary Gland. *Cell Stem Cell* **20**, 247-260.e245, doi:10.1016/j.stem.2016.11.007 (2017).

REVIEWERS' COMMENTS

Reviewer #1 (Remarks to the Author):

I appreciate the efforts the authors made to address my questions. Although the new results obtained raised more questions than answering them.

What puzzling me the most is why would a senescent cell highly proliferative? Authors commented that senescence should and could not be defined by simple markers, which I agree 100%. However, would transcriptome analysis be sufficient when the same cells are highly proliferative?

Anyway, we might not share the same definition of what a senescence state is. At the point, we just have to accept that some questions could not be answered in this manuscript at this stage.

What puzzling me the most is why would a senescent cell highly proliferative? Authors commented that senescence should and could not be defined by simple markers, which I agree 100%. However, would transcriptome analysis be sufficient when the same cells are highly proliferative?

Our response:

We normally regard 'senescence' as a certain static cellular state associated with cell cycle arrest. However, increasing evidence suggest that the senescent cell cycle arrest may not be terminal, in certain conditions, senescence cells can reenter cell cycle [1-3]; in addition, senescent features are heterogeneous including aberrant epigenetics, abnormal secretome, functional decline etc. Cell-cycle arrest is one of the features, but not the only feature. Senescence is also a dynamic process showing different features at different stages, which has been reviewed [4]. According to our data, cell cycle arrest is a feature of early senescent cells, at later senescence stage, these cells acquire ability to reenter cell cycle.

1. *Beausejour, C. M. et al. Reversal of human cellular senescence: roles of the p53 and p16 pathways. EMBO J. 22, 4212–4222 (2003).*
2. *17. Sage, J., Miller, A. L., Perez-Mancera, P. A., Wysocki, J. M. & Jacks, T. Acute mutation of retinoblastoma gene function is sufficient for cell cycle re-entry. Nature 424, 223–228 (2003)*
3. *Milanovic, M., et al., Senescence-associated reprogramming promotes cancer stemness. Nature, 2018. 553(7686): p. 96-100.*
4. *Lee, S. and C.A. Schmitt, The dynamic nature of senescence in cancer. Nat Cell Biol, 2019. 21(1): p. 94-101.*
